# Supplementary material for: Tissue-Restricted Adaptive Type 2 Immunity Is Orchestrated by Expression of the Costimulatory Molecule OX40L on Group 2 Innate Lymphoid Cells
Source: Immunity. 2018 Jun 19;48(6):1195–1207.e6. doi: 10.1016/j.immuni.2018.05.003 (PMC6015114; doi:10.1016/j.immuni.2018.05.003)
Supplement: Document S1. Figures S1–S7 [file mmc1.pdf]

Immunity, Volume *48*

Supplemental Information

Tissue-Restricted Adaptive Type 2 Immunity Is Orchestrated by Expression of the Costimulatory Molecule OX40L on Group 2 Innate Lymphoid Cells

Timotheus Y.F. Halim, Batika M.J. Rana, Jennifer A. Walker, Bernhard Kerscher, Martin D. Knolle, Helen E. Jolin, Eva M. Serrao, Liora Haim-Vilmovsky, Sarah A. Teichmann, Hans-Reimer Rodewald, Marina Botto, Timothy J. Vyse, Padraic G. Fallon, Zhi Li, David R. Withers, and Andrew N.J. McKenzie

Supplemental Figures and Figure Legends

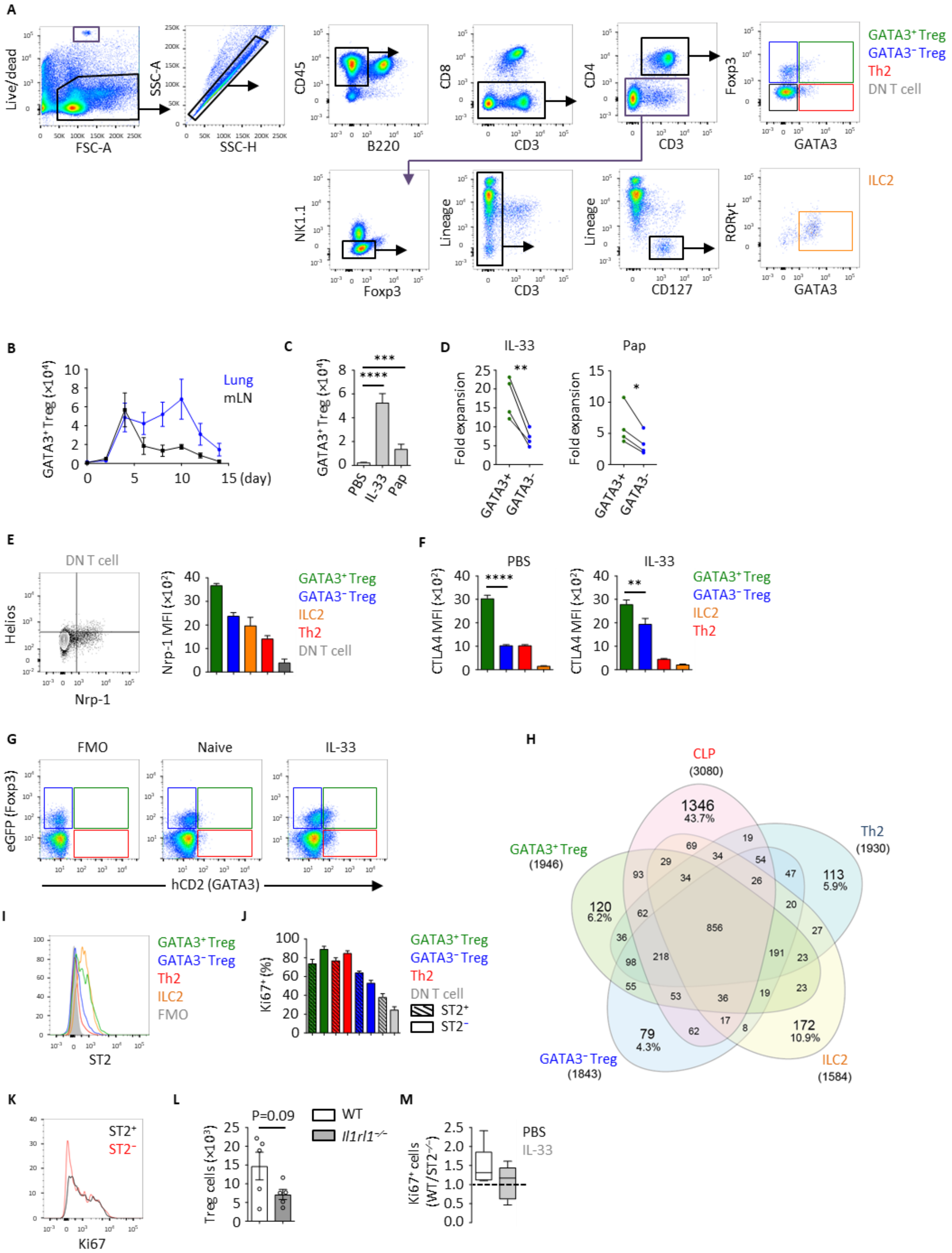

Figure S1 (Related to Figure 1)

A) A representative flow cytometry gating strategy for identifying and analysing adaptive type-2 lymphocytes. Live cells were identified by fixable viability dye exclusion. CD45^{+}B220^{-}CD8^{-}CD3^{+}CD4^{+} (CD4) T cells were gated based on intracellular expression of Foxp3 and GATA3. CD45^{+}B220^{-}CD8^{-}CD3^{-}CD4^{-}NK1.1^{-}Lineage^{-}CD127^{+} innate lymphoid

cells (ILC) were gated based on expression of RORγt and GATA3. These cells were subsequently analysed for other surface or intracellular markers;

B) WT mice were exposed to papain (i.n., day 0 and 1) followed by quantification of $GATA3^{+}$ $Foxp3^{+}$ Treg cells in the lung and mediastinal lymph node (mLN).

C) WT mice were exposed to papain (Pap) or IL-33 (i.n., day 0 and 1) followed by quantification of $GATA3^{+}Foxp3^{+}$ Treg cells in the lung on day 5.

D) Fold expansion of $GATA3^{+}Foxp3^{+}$ and $GATA3^{+}Foxp3^{+}$ Treg cells in the lungs on day 5 of IL-33 and papain (Pap) treated mice (i.n., day 0 and 1). Fold expansion over PBS treated control groups was measured for each experiment (4-6 mice per group, average values of 4 individual experiments are shown, paired groups for each individual experiment are shown).

E) The indicated populations (see **Fig S1A**) were analysed for expression of Helios and Nrp-1. Mean fluorescence intensity of Nrp-1 was measured. Flow cytometry data for DN T cells ($Foxp3^{-}GATA3^{-}$, see a) is shown.

F) The indicated populations (see **Fig S1A**) were analysed for intracellular staining for CTLA4 on day 5 in PBS or IL-33 treated mice (i.n., day 0 and 1).

G) Lung ILC2, $GATA3^{+}$ and $GATA3^{-}$ Treg and Th2 cells were FACS purified on day 5 from *Foxp3*$^{EGFP\text{-}DTR/+}$*Gata3*$^{hCD2/+}$ mice treated with PBS or IL-33 (i.n., day 0 and 1). Shown is the gating profile of eGFP (*Foxp3*) and anti-hCD2 (*Gata3*) staining, and the FMO control.

H) Gene expression of naïve $Foxp3^{egfp+}GATA3^{hDC2+}$ and $Foxp3^{egfp+}GATA3^{hDC2-}$ Treg, $Foxp3^{egfp-}GATA3^{hDC2+}$ Th2 cell, ILC2 and common lymphoid progenitor (CLP) was compared. Shown is a Venn diagram of transcripts expressed in each cell population (> 10 RPKM).

I) ST2 expression was measured on lung ILC2, $GATA3^{+}$ and $GATA3^{-}$ Treg and Th2 cells in WT mice on day 5 after treatment with IL-33 (i.n., day 0 and 1).

J) The indicated populations from mouse lungs (see **Fig S1A**) were analysed on day 5 for the expression of ST2 and intracellular Ki67, after treatment with IL-33 (i.n., days 0 and 1). $ST2^{+}$ and $ST2^{-}$ fractions were analysed for percentage of dividing ($Ki67^{+}$) cells.

K) WT mouse lung $ST2^{+}$ and $ST2^{-}$ Treg cells were stained for intracellular Ki67 expression on day 5 after IL-33 treatment (i.n., day 0 and 1).

L-M) Splenic $CD4^{+}$ T cells from (CD45.1) WT and (CD45.2) *Il1rl1*$^{-/-}$ mice were enriched, and injected in to *Rag2*$^{-/-}$ mice at a 50:50 ratio (i.v.). These recipients were treated with PBS or IL-33 (i.n., days 0 and 1), followed by quantification of $Ki67^{+}$ CD45.1 and CD45.2 $CD4^{+}$ $Foxp3^{+}$ Treg cells in the lungs on day 5 (IL-33 treated shown, **L**). The ratio of $Ki67^{+}$ lung Treg cells in *Il1rl1*$^{-/-}$ over WT was calculated to establish if one had a selective advantage to proliferate in either PBS or IL-33 treated animals **(M)**.

Bar graphs indicate mean (± S.E.M). (**A**, general representation of gating), (**B**, n=4,4 at each time point, mean ±S.E.M., 2 repeat experiments), (**C**, n=4,4,4 left to right, ANOVA, 2 repeat experiments), (**E**, n=4,4,4,4 left to right, 3 repeat experiments), (**F**, n=4,4,4,4 left to right, ANOVA, 3 repeat experiments), (**G**, general representation of gating), (**H**, n=2, single experiment), (**I-K**, n=4 in all, general representation of gating shown, 2 repeat experiments), (**L-M**, n=5,5 left to right, two-tailed Student's t-test, 2 repeat experiments), ** = $p \leq 0.01$, *** = $p \leq 0.001$, **** = $p \leq 0.0001$.

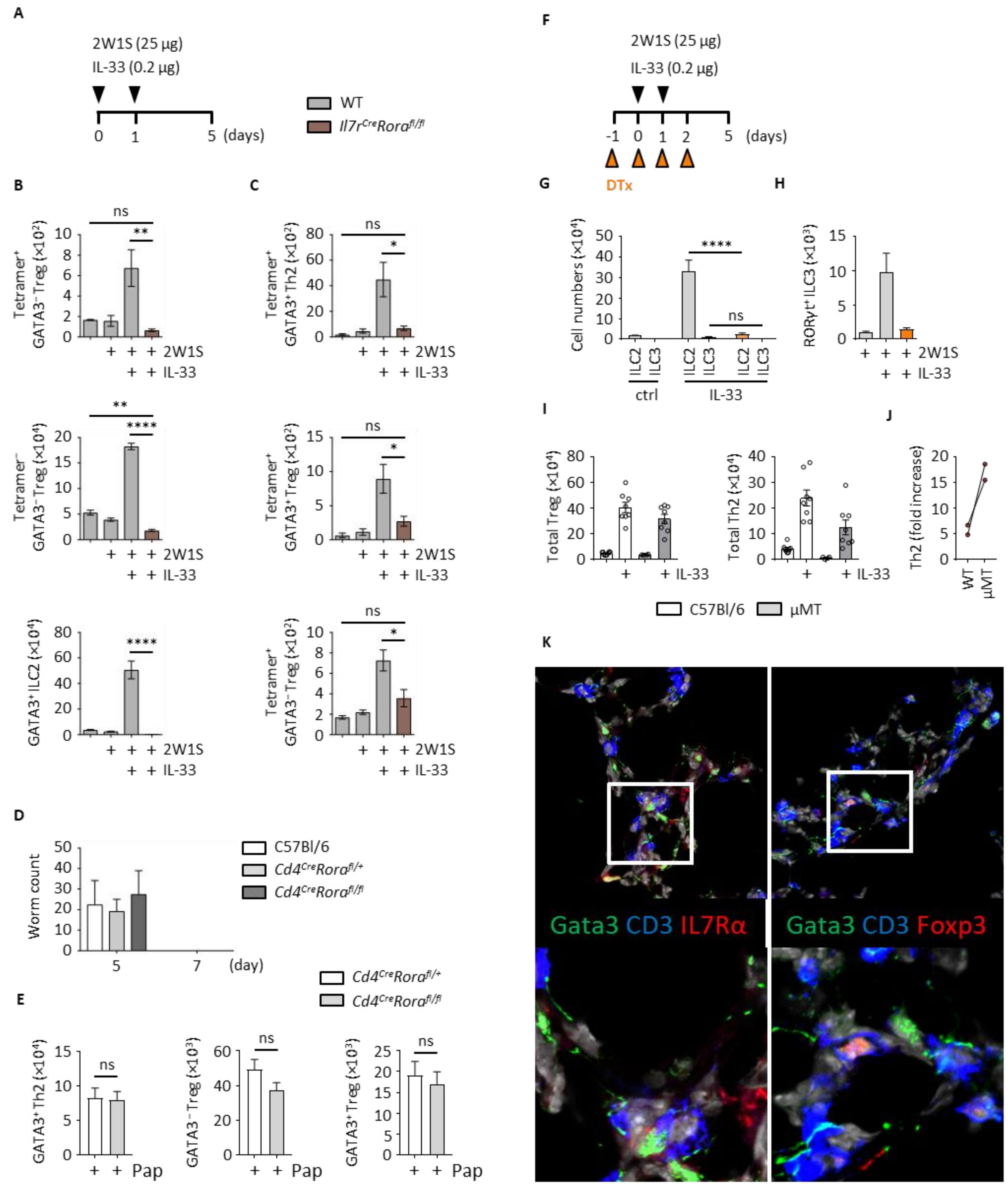

Figure S2 (Related to Figure 1)

A-C) WT and ILC2-deficient mice (*Il7r*^{Cre}*Rora*$^{fl/fl}$) were injected with 2W1S-peptide and IL-33 as indicated (i.n., day 0 and 1). Lung and mLN cells were collected on day 5, followed by analysis and quantification of 2W1S:Tetramer^{+} and 2W1S:Tetramer^{-} CD4^{+} T cell populations and lung ILC2 (**B**) and mLN (**C**)

D) Mice of the indicated genotypes were infected with *Nippostrongylus brasiliensis* (500 L3 larvae, subcutaneously, day 0) followed by quantification of worms in the small intestines on day 5 and 7.

E) Mice of the indicated genotypes were injected with papain (Pap) (i.n., day 0, 1 and 22 or 23). Lung cells were collected on day 27 or 28, followed by analysis and quantification of Th2, GATA3$^+$ Treg, and GATA3$^-$ Treg cell populations.
F-H) ILC2-depleted (DTx) and non-depleted ICOS-T (PBS) mice (Oliphant et al., 2014) were injected with 2W1S-peptide and IL-33 as indicated (i.n., day 0 and 1). Lung and mLN cells were collected on day 5, followed by analysis and quantification of 2W1S:Tetramer$^+$ and 2W1S:Tetramer$^-$ CD4$^+$ T cell populations and lung ILC2 and Live CD45$^+$B220$^-$CD3ε$^-$CD4/8$^-$NK1.1$^-$Lin$^-$CD127$^+$GATA3$^-$RORγt$^+$ ILC3 (**G and H**).
I-J) Mice of the indicated genotypes were injected with IL-33 on days 0 and 1, followed by analysis on day 5 of total lung Treg and Th2 cell number (**I**), and the fold increase of Th2 cells in each genotype (**J**). Average fold increase is shown for two independent experiments.
K) Frozen sections from IL-33 treated (i.n., day 0 and 1) mouse lungs, collected on day 5, were stained for CD3, GATA3, and IL7Rα or Foxp3 as indicated. Bottom panel shows the gated region in the upper panel. Scale bar denotes 50 µm. Data are representative of at least 2 independent experiments.

Bar graphs indicate mean (± S.E.M). (**B-C**, n=4,4,4,4 left to right, ANOVA, 3 repeat experiments), (**D**, n=4,7,6,4,7,6 left to right, ANOVA, 2 similar repeat experiments), (**E**, n=10,10 left to right for all panels, two-tailed Student's t-test, 2 pooled experiments shown), (**G**, n=4,4,4 (for both ILC2 and ILC3) left to right, ANOVA, 3 repeat experiments), (**H**, n=4,4,4 left to right, 3 repeat experiments), (**I**, n=8,8,8 left to right, ANOVA, pooled data of 2 repeat experiments), (**J**, data points represent mean fold increase per genotype, 2 independent experiments), ns = not significant, * = p≤0.05, ** = p≤0.01, **** = p≤0.0001.

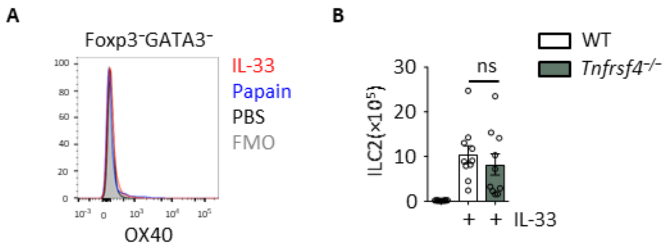

Figure S3 (Related to Figure 2)

A) OX40 expression was analysed on CD4$^+$ Foxp3$^-$GATA3$^-$ DN T cells from mice treated with PBS, papain (Pap) or IL-33. Fluorescence minus one (FMO) control was used to establish gating threshold.
B) Lung ILC2 cells were quantified on day 5 post IL-33 treatment (i.n.) in PBS and IL-33 treated WT and *Tnfrsf4*$^{-/-}$ mice.

Bar graphs indicate mean (± S.E.M). (**A**, general representation of gating), (**B**, n=10,10,10 left to right, ANOVA, 2 pooled repeat experiments), ns = not significant.

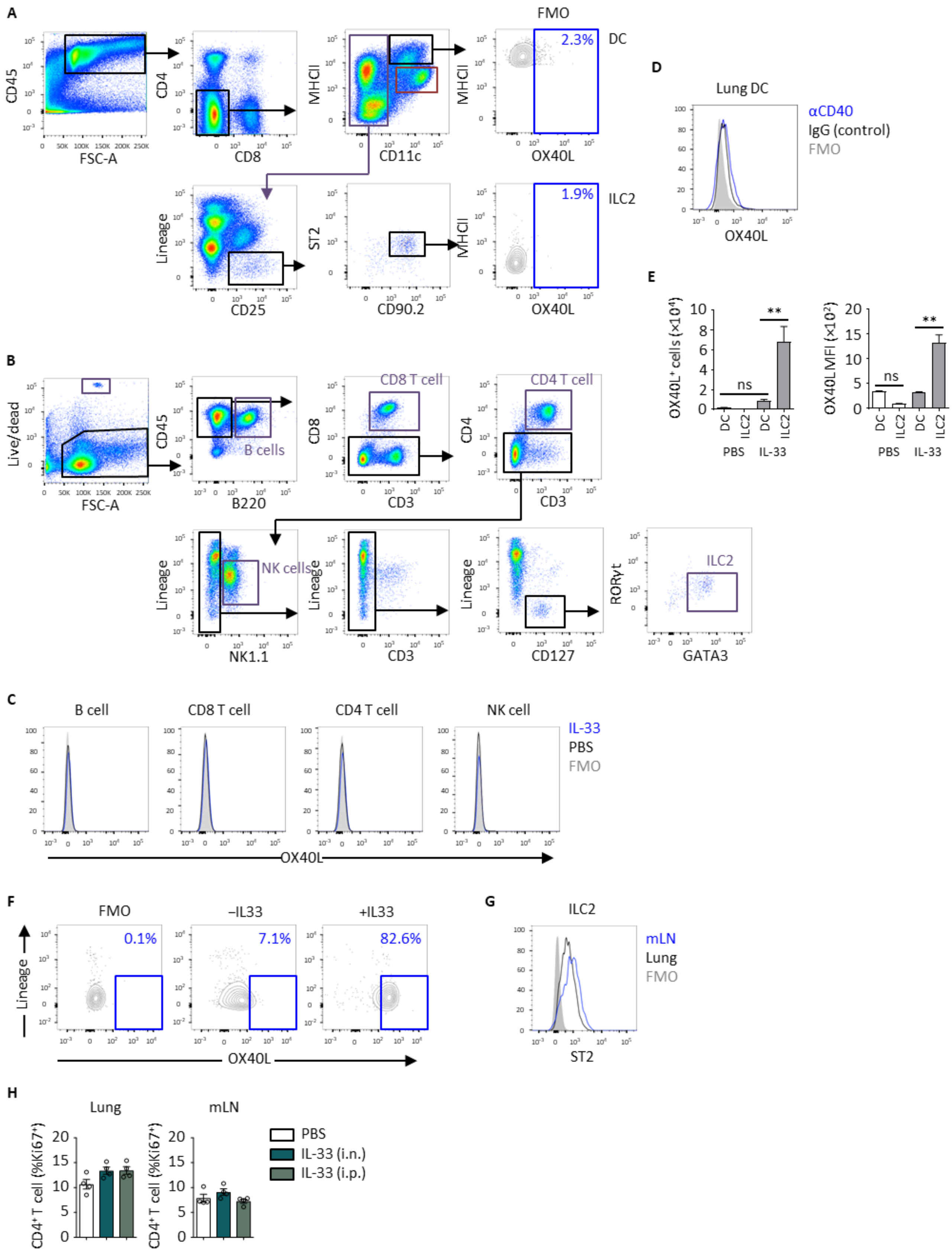

Figure S4 (Related to Figure 3)

A-B) OX40L expression on different immune cells was established by flow cytometry analysis. Lung cells from WT mice were gated for $CD45^+$ leukocytes and dead cells were excluded (not shown), followed by the indicated gating strategies to identify dendritic cells (DC), macrophages (MΦ), ILC2, ILC3, natural killer (NK) cells, B cells, CD8 and CD4 T cells. Fluorescence minus one (FMO) controls were used to set the gate for OX40L expression.
C) OX40L expression was measured on the indicated immune cells in WT mice on day 2 after stimulation with PBS or IL-33 (i.n., day 0 and 1).
D) Mice were administered anti-CD40 (αCD40) or IgG control mAb on days 0 and 1, followed by analysis of lung DCs for OX40L expression on day 2.
E) The absolute number of $OX40L^+$ ILC2 and DC in the lungs was calculated in PBS or IL-33 stimulated mice on day 3 (left panel). Mean fluorescence intensity (MFI) of OX40L staining was calculated for ILC2 and DC in the lungs of PBS or IL-33 stimulated mice on day 3 (right panel).
F) Human blood ILC2 were purified by FACS, followed by stimulation in culture with IL-2 and IL-7 (20 ng/ml) ± IL-33 (20 ng/ml) for 3 days. On day 3, cells were analysed for expression of OX40L on $CD45^+lineage^-$ ILC2.
G) ST2 expression was analysed on mLN and Lung ILC2 ($CD45^+B220^-CD4^-CD8^-$ $CD3^-NK1.1^-Lineage^-CD25^+GATA3^+$) in mice treated as in **Fig 3E**. Fluorescence minus one (FMO) control was used to indicate background staining.
H) WT mice received IL-33 via intranasal or intraperitoneal route (day 0 and 1, PBS control), followed by analysis (day 2) of lung and mLN %$Ki67^+$ $CD4^+$ T cells.

Bar graphs indicate mean (± S.E.M). (**A-C**, general representation of gating, 2-3 repeat experiments), (**D**, representative histogram, 2 repeat experiments), (**E**, n=3,3,3,3 left to right, ANOVA, 2 repeat experiments), (**F**, single individual sorted ILC2 exposed to different *in vitro* conditions shown, percent gated populations shown, 2 repeat experiments), (**G**, representative histogram, 3 repeat experiments), (**H,** n=4,4,4 left to right, ANOVA, 2 repeat experiments), ns = not significant, ** = p≤0.01.

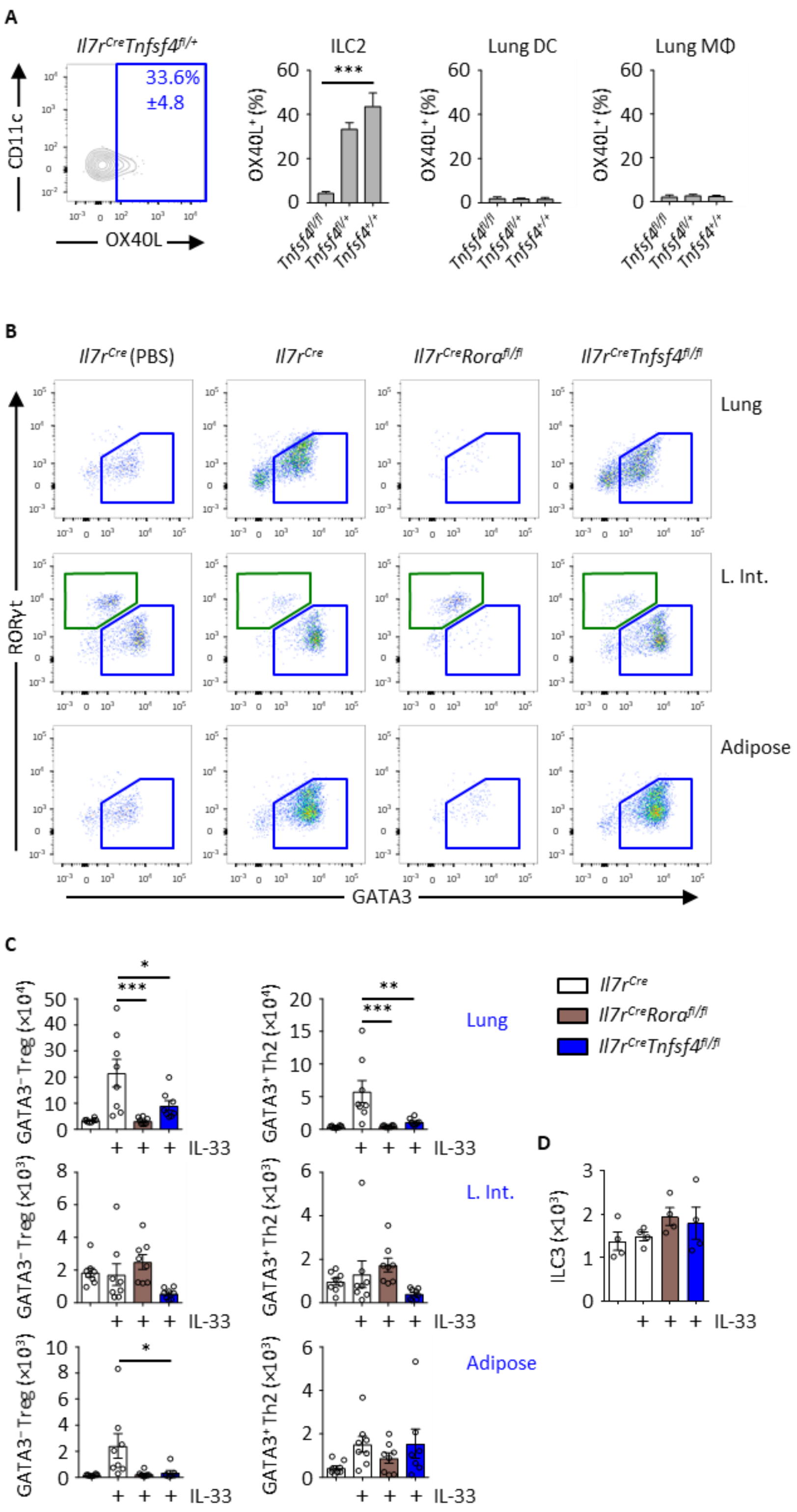

Figure S5 (Related to Figure 4)

A) OX40L expression was measured on lung ILC2, DC and MΦ from *Il7r*^{Cre}, *Il7r*^{Cre}*Rora*$^{fl/fl}$ and *Il7r*^{Cre}*Tnfsf4*$^{fl/fl}$ mice on day 2 after IL-33 administration (i.n., day 0 and 1). FMO control was used to establish gating threshold.

B) Mice of the indicate genotypes were analysed for ILC2 (blue) and ILC3 (green) in the indicated tissues (L. int., large intestine) on day 5 (as in **Fig S1A**) after treatment with PBS or

IL-33 (i.p., day 0 and 1). Total ILC2 were quantified, and are displayed on the right. Adipose cell numbers are normalised to cells/mg of tissue.

C-D) Mice of the indicate genotypes were analysed for GATA3^{-} Treg and Th2 cells in the indicated tissues on day 5 (as in **Fig S1A**) (**C**), and ILC3 (**D**) after treatment with PBS or IL-33 (i.p., day 0 and 1).

Bar graphs indicate mean (± S.E.M). (**A**, right panel shows percent gated ± S.D., n=4, left bar graphs n=4,3,4 left to right, 2 repeat experiments). (**B**, right panel shows general representation of gating, 2 repeat experiments). (**C**, n=8,8,8,8 left to right, ANOVA, 2 pooled repeat experiments, (n=8,8,8,7 for Adipose)), (**D**, n=4,4,4,4 left to right, 2 repeat experiments), * = p≤0.05, ** = p≤0.01, *** = p≤0.001.

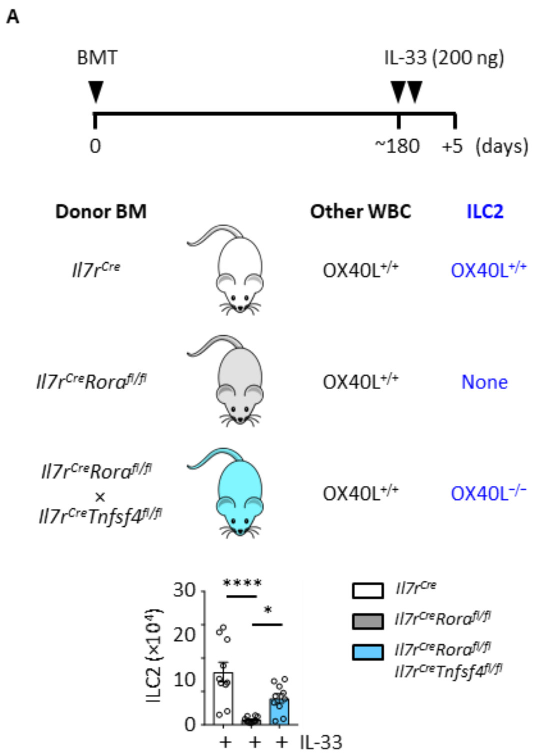

Figure S6 (Related to Figure 5)

A) Bone marrow and mixed-bone marrow (BM) chimeric animals were created with the indicated genotypes. Bone marrow transfer (BMT) was performed on day 0 after lethal irradiation (9.0 Gy). 6 to 7 months after bone marrow transfer, mice received IL-33 (i.n., days 0 and 1) followed by quantification of lung ILC2 cells on day 5.

Bar graphs indicate mean (± S.E.M). (**A**, n=10,10,10 left to right, ANOVA, 2 pooled repeat experiments), * = p≤0.05, **** = p≤0.0001.

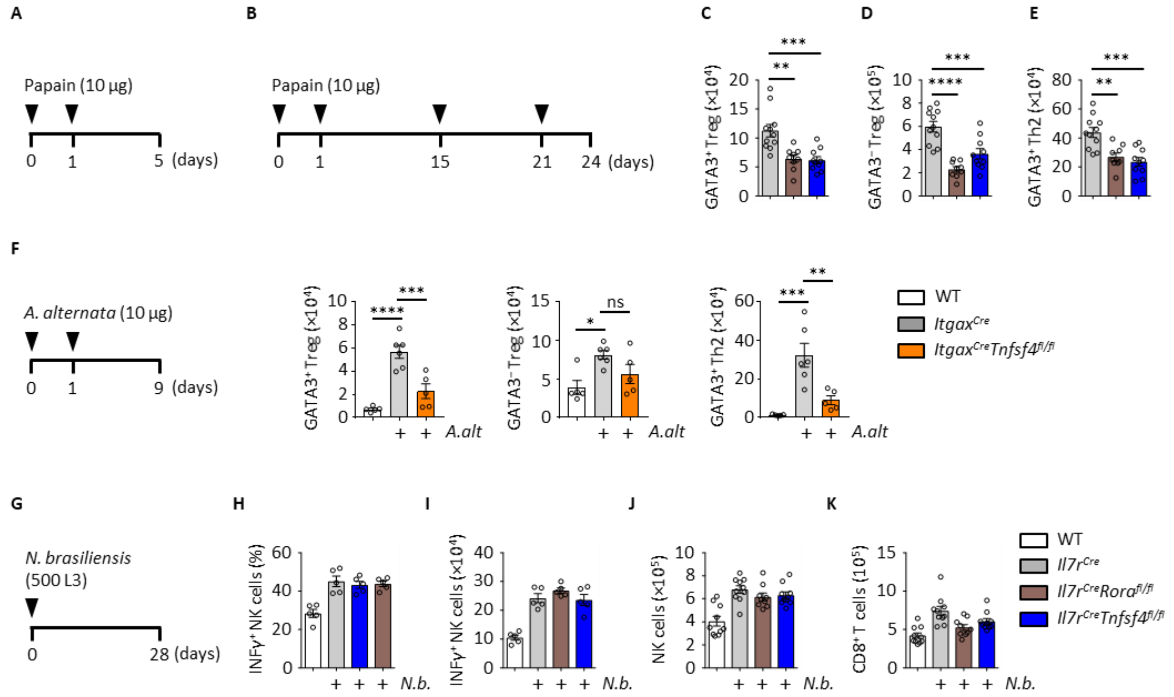

Figure S7 (Related to Figure 6 and 7)

A-B) Mice were treated with PBS or papain (Pap) as indicated, followed by analysis on day 5 or 24.

C-E) Mice of the specified genotypes were treated with papain (Pap) on days 0, 1, 14 and 21, followed by quantification on day 24 of lung Th2, GATA3$^+$ Treg, and GATA3$^-$ Treg cells.

F) Mice of the specific genotypes were injected with *A. alternata* (*A.alt*) on days 0 and 1 (i.n.), followed by analysis on day 9 of lung Th2, GATA3$^+$ Treg, and GATA3$^-$ Treg cells.

G) Mice of the specified genotypes were infected with *Nippostrongylus brasiliensis* (*N.b.*) on day 0, followed by analysis on day 28 of:

(H-I) Whole lung cell-suspensions were re-stimulated with PMA and ionomycin, followed by identification of IFNγ$^+$ NK cells by intracellular staining. Percent and total IFNγ$^+$ NK cells positive cells are shown.

J) Total lung NK cells numbers were quantified.

K) Total CD8$^+$ T cell numbers were quantified.

Bar graphs indicate mean (± S.E.M). (**C-E**, n=11,9,10 left to right, ANOVA, 3 repeat experiments (2 pooled experiments shown)), (**F**, n=5,5,5 left to right, 2 repeat experiments), (**H** and **I**, n=5,5,5,5 left to right, ANOVA, 2 repeat experiments), (**J** and **K**, n=10,10,10,10 left to right, ANOVA, 3 repeat experiments (2 pooled experiments shown)), ns = not significant, * = p≤0.05, ** = p≤0.01, *** = p≤0.001, **** = p≤0.0001.
