# Supplementary material for: Tissue-Restricted Adaptive Type 2 Immunity Is Orchestrated by Expression of the Costimulatory Molecule OX40L on Group 2 Innate Lymphoid Cells
Source: Immunity. 2018 Jun 19;48(6):1195–1207.e6. doi: 10.1016/j.immuni.2018.05.003 (PMC6015114; doi:10.1016/j.immuni.2018.05.003)
Supplement: Document S2. Article plus Supplemental Information [file mmc4.pdf]

Immunity

Article

Tissue-Restricted Adaptive Type 2 Immunity Is Orchestrated by Expression of the Costimulatory Molecule OX40L on Group 2 Innate Lymphoid Cells

Graphical Abstract

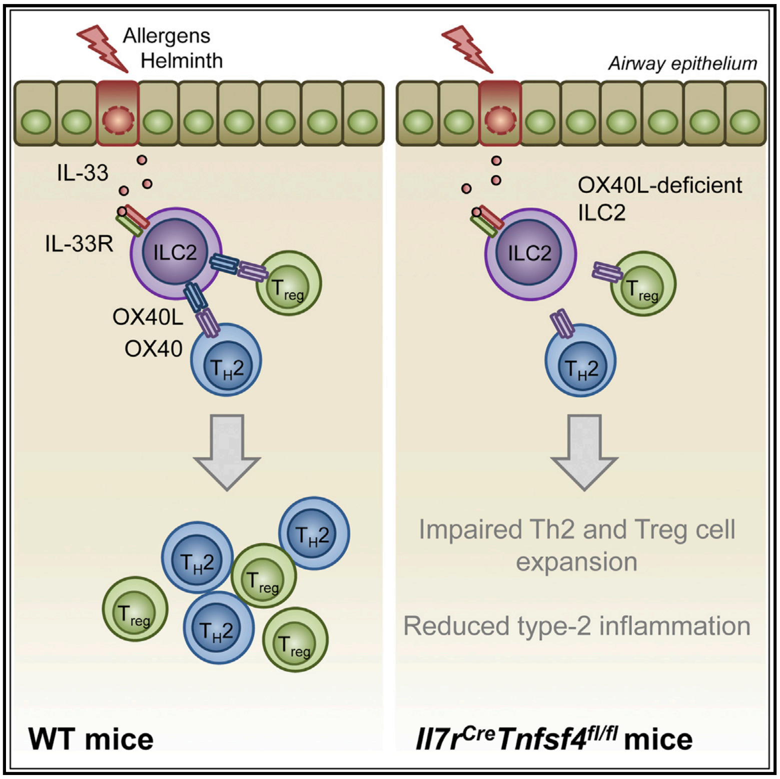

Highlights

- Tissue-specific ILC2s are the primary source of OX40L after IL-33 stimulation
- ILC2s from *Il7r*^{Cre}*Tnfsf4*$^{fl/fl}$ mice do not express OX40L upon IL-33 stimulation
- OX40L expression by ILC2s is required for IL-33-driven Th2 and Treg cell expansion
- *Il7r*^{Cre}*Tnfsf4*$^{fl/fl}$ mice have impaired Th2 and Treg cell responses

Authors

Timotheus Y.F. Halim,
Batika M.J. Rana,
Jennifer A. Walker, ..., Zhi Li,
David R. Withers,
Andrew N.J. McKenzie

Correspondence

tim.halim@cruk.cam.ac.uk (T.Y.F.H.),
anm@mrc-lmb.cam.ac.uk (A.N.J.M.)

In Brief

Type 2 immunity underpins diverse processes central to tissue homeostasis, allergic inflammation, and anti-helminth immunity. Halim et al. demonstrate that the local expansion of Th2 and Treg cells in response to the alarmin IL-33 is dependent on the expression of the costimulatory molecule OX40L by type 2 innate lymphoid cells (ILC2s), revealing a central role for the IL-33-ILC2-OX40L pathway in the orchestration of type 2 immunity.

Halim et al., 2018, Immunity *48*, 1195–1207
June 19, 2018 © 2018 MRC Laboratory of Molecular Biology.
Published by Elsevier Inc.
https://doi.org/10.1016/j.immuni.2018.05.003

CellPress

Tissue-Restricted Adaptive Type 2 Immunity Is Orchestrated by Expression of the Costimulatory Molecule OX40L on Group 2 Innate Lymphoid Cells

Timotheus Y.F. Halim,[1,2,11,*] Batika M.J. Rana,[1] Jennifer A. Walker,[1] Bernhard Kerscher,[1,10] Martin D. Knolle,[1,3] Helen E. Jolin,[1] Eva M. Serrao,[2] Liora Haim-Vilmovsky,[4] Sarah A. Teichmann,[4] Hans-Reimer Rodewald,[5] Marina Botto,[6] Timothy J. Vyse,[7] Padraic G. Fallon,[8] Zhi Li,[9] David R. Withers,[9] and Andrew N.J. McKenzie[1,*]
[1]MRC Laboratory of Molecular Biology, Cambridge CB2 0QH, UK
[2]University of Cambridge, CRUK Cambridge Institute, Cambridge CB2 0RE, UK
[3]Department of Medicine, University of Cambridge, Cambridge CB2 0QQ, UK
[4]Wellcome Sanger Institute, Wellcome Genome Campus, Hinxton CB10 1SA, UK
[5]Division of Cellular Immunology, German Cancer Research Center, Heidelberg 69120, Germany
[6]Imperial College London, Department of Medicine, London, UK
[7]King's College London, Department of Medical and Molecular Genetics, London, UK
[8]Trinity Biomedical Sciences Institute, Trinity College Dublin, Ireland
[9]University of Birmingham, Institute of Immunology and Immunotherapy, Birmingham B15 2TT, UK
[10]Present address: Division of Microbiology, Paul-Ehrlich-Institut, Langen, Germany
[11]Lead Contact
*Correspondence: tim.halim@cruk.cam.ac.uk (T.Y.F.H.), anm@mrc-lmb.cam.ac.uk (A.N.J.M.)
https://doi.org/10.1016/j.immuni.2018.05.003

SUMMARY

The local regulation of type 2 immunity relies on dialog between the epithelium and the innate and adaptive immune cells. Here we found that alarmin-induced expression of the co-stimulatory molecule OX40L on group 2 innate lymphoid cells (ILC2s) provided tissue-restricted T cell co-stimulation that was indispensable for Th2 and regulatory T (Treg) cell responses in the lung and adipose tissue. Interleukin (IL)-33 administration resulted in organ-specific surface expression of OX40L on ILC2s and the concomitant expansion of Th2 and Treg cells, which was abolished upon deletion of OX40L on ILC2s (*Il7ra*$^{Cre/+}$*Tnfsf4*$^{fl/fl}$ mice). Moreover, *Il7ra*$^{Cre/+}$ *Tnfsf4*$^{fl/fl}$ mice failed to mount effective Th2 and Treg cell responses and corresponding adaptive type 2 pulmonary inflammation arising from *Nippostrongylus brasiliensis* infection or allergen exposure. Thus, the increased expression of OX40L in response to IL-33 acts as a licensing signal in the orchestration of tissue-specific adaptive type 2 immunity, without which this response fails to establish.

INTRODUCTION

Type 2 immunity underpins numerous key homeostatic and immune processes in health and disease (Pulendran and Artis, 2012). The type 2 cytokine environment is regulated at the tissue level by the release of epithelium-derived type 2 cytokines, including interleukin (IL)-25, thymic stromal lymphopoietin (TSLP), and the alarmin IL-33. These molecules elicit the production of type 2 effector cytokines from immune cells, which are fundamental for diverse functions, ranging from anti-helminth parasite immunity to allergic inflammation, wound healing responses, and metabolism (Gause et al., 2013; Man et al., 2017). Indeed, the tissue- and context-specific production of type 2 alarmins likely governs their downstream physiological functions. Adaptive type 2 immunity is driven by CD4$^+$ T helper 2 (Th2) cells, which are the major source of type 2 inflammatory cytokines. However, CD4$^+$ regulatory T (Treg) cells are increasingly associated with some type 2 inflammatory functions, such as wound healing and adipose tissue homeostasis (Odegaard and Chawla, 2015; Panduro et al., 2016). The nature of the Treg cell association with type 2 immunity remains enigmatic and ranges from suppressive to synergistic. Indeed, Th2 and Treg cell subsets can respond directly to type 2 alarmins such as IL-33 and express overlapping transcriptional and functional programs (Siede et al., 2016; Wohlfert et al., 2011). While adaptive type 2 immune cells are rare in most tissues under homeostatic conditions, innate group 2 innate lymphoid cells (ILC2s) are tissue-resident cells and rapidly respond to type 2 alarmins by producing factors shared with both Th2 (IL-5 and IL-13) and Treg (Amphiregulin) cell function. The division of labor, and interactions, between ILC2s and adaptive type 2 immune cells remains a fundamental and unresolved question.

Roles for ILC2s directly regulating CD4$^+$ T helper cell activation have been proposed recently (Drake et al., 2014; Mirchandani et al., 2014; Oliphant et al., 2014; von Burg et al., 2014). MHCII-expressing ILC2s interact with antigen-specific T cells to instigate a dialog in which ILC2 and T cell crosstalk contributes to their mutual maintenance, expansion, and cytokine production (Mirchandani et al., 2014; Oliphant et al., 2014). ILC2s can also modulate T cell function, not by acting directly on naive CD4$^+$ T cells but by collaborating with dendritic cells (DCs) to induce Th2 cell activation (Halim et al., 2014, 2016). Notably,

This is an open access article under the CC BY license (http://creativecommons.org/licenses/by/4.0/).

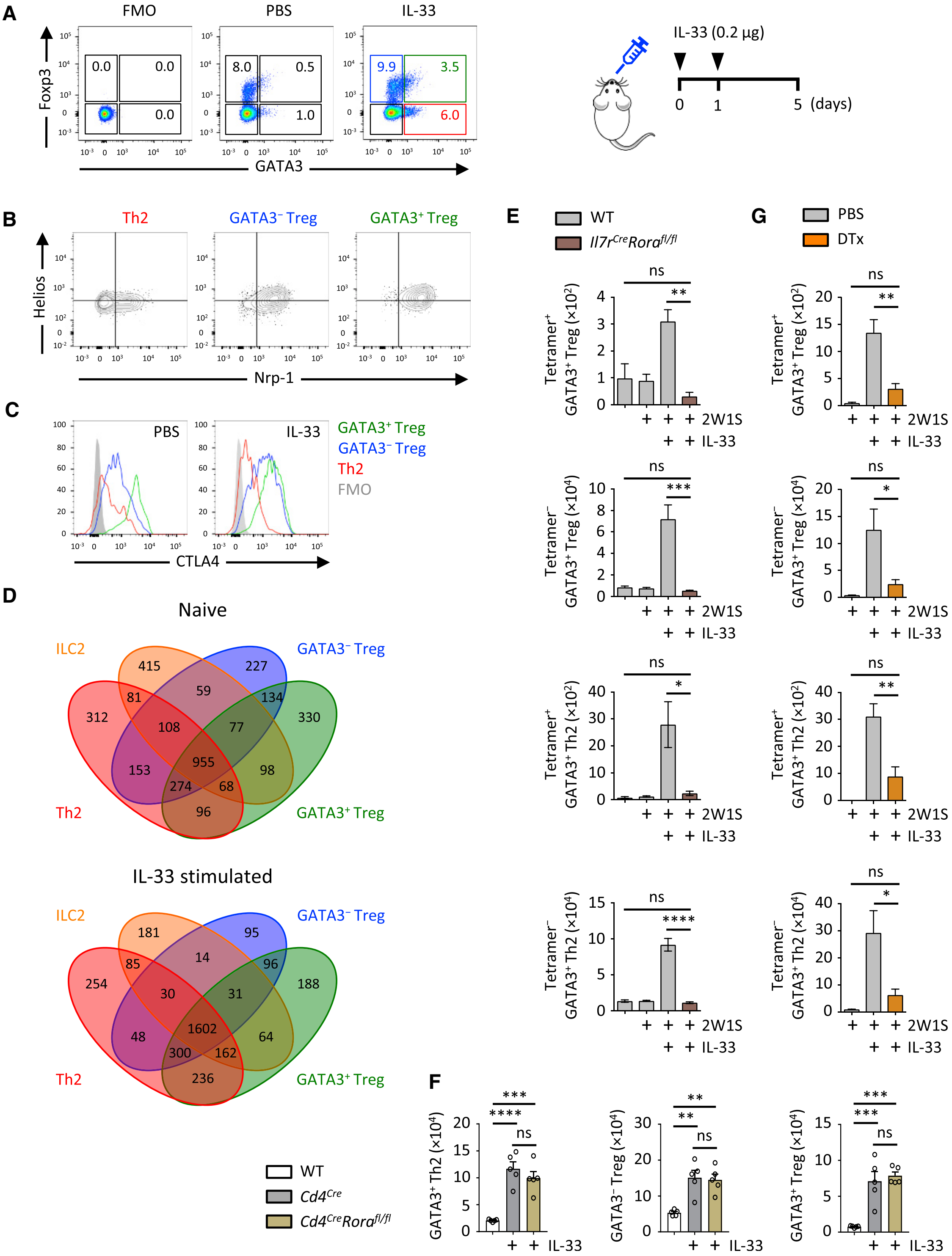

Figure 1. ILC2s Are Required for Th2 and Treg Cell Response to IL-33

(A–C) WT mice were treated with PBS or IL-33 (i.n., day 0 and 1) and analyzed on day 5 for Foxp3 and GATA3 expression in lung $CD4^+$ T cells (A). Indicated populations (IL-33-treated shown) were subsequently analyzed for expression of Helios and neuropilin-1 (Nrp-1) (B) and CTLA4 (C).

(D) RNA-seq was performed on lung $Foxp3^{egfp+}GATA3^{hDC2+}$ and $Foxp3^{egfp+}GATA3^{hDC2-}$ Treg cells, $Foxp3^{egfp-}GATA3^{hDC2+}$ Th2 cells, and ILC2s on day 5 after treatment with PBS or IL-33 (i.n., day 0 and 1). Shown is a Venn diagram of transcripts expressed in each cell population (>10 RPKM).

(E) Mice were treated with IL-33 and 2W1S-peptide as indicated (i.n., day 0 and 1), followed by quantification of 2W1S-$Tetramer^{+/-}$ $Foxp3^+GATA3^+$ Treg cells and $Foxp3^-GATA3^+$ Th2 cells in the lung on day 5.

(legend continued on next page)

the importance of the local tissue environment is highlighted by the observation that the response to systemic antigen exposure is not sensitive to the absence of ILC2s (Gold et al., 2014). In contrast to these models where ILC2 help initiates and maintains type 2 responses, it has been proposed that ILC2 and $CD4^+$ T cell responses develop independently and that it is the tissue-localized exposure of these cells to locally produced cytokines that acts as a checkpoint in the activation of a type 2 response, rather than the interaction of these cells within the tissue (Van Dyken et al., 2016). Thus, the interplay between ILC2s and adaptive type 2 immune cells is likely to differ depending on anatomical location, activating signals, and temporal phase of the immune response.

In addition to the MHCII-mediated activation of helper T cells, the cognate interaction between the co-stimulatory molecule ICOSL on ILC2s and ICOS on T cells promotes Treg cell accumulation following IL-33 administration (Molofsky et al., 2015). This is in addition to the ICOS-ICOSL autocrine signals that can enhance ILC2 proliferation (Maazi et al., 2015). An *in vitro* study also suggested the potential contribution for OX40 ligand (OX40L) expressed on ILC2s for the co-stimulation of T cells, though its role *in vivo* was not explored (Drake et al., 2014). Ligation of OX40 by OX40L, encoded respectively by the genes *Tnfrsf4* and *Tnfsf4*, provides an important signal for the expansion or survival of Th2 cells, while less is understood about these effects on Treg cells (Croft, 2010; Webb et al., 2016). OX40 is constitutively expressed at baseline on Treg cells and is induced on Th2 cells after T cell receptor (TCR)-mediated activation, while OX40L expression is reported on numerous immune cells but most notably on professional antigen-presenting cells such as DCs. Specifically, OX40L expression by DCs is important for effective Th2 cell responses to helminth-derived antigen (Jenkins et al., 2007). OX40 binding to OX40L activates TNF receptor associated factor (TRAF) signaling pathways that synergize with TCR or cytokine-derived stimulation (Croft, 2010; Webb et al., 2016).

Here we found that ILC2s express high levels of OX40L after exposure to IL-33. No other immune cells that we analyzed in the lung, including lung DCs, expressed OX40L *in vivo* in response to exogenously administered type 2 alarmins, including TSLP. OX40L expression on ILC2s was tissue restricted and correlated with local expansion of adaptive type 2 immune cells. ILC2s and OX40 were critical for tissue-specific IL-33-driven Th2 and Treg (preferentially $GATA3^+$ Treg) cell responses. ILC2-targeted deletion of OX40L ($Il7r^{Cre/+}Tnfsf4^{fl/fl}$) significantly impaired Th2 and Treg cell expansion following allergen exposure and *Nippostrongylus brasiliensis* helminth infection, with profound effects on overall type 2 inflammation. Thus, OX40L expression on ILC2s in response to epithelial cell-derived alarmins is a critical checkpoint for orchestrating adaptive type 2 responses.

RESULTS

ILC2 Are Critical for Regulating Adaptive Type 2 Immunity

Airway exposure to the protease allergen papain results in IL-33-dependent accumulation of $GATA3^+$ ILC2s and Th2 cells. The transcription factor GATA3 is critical for the development and function of type 2 cytokine-producing ILC2s and Th2 cells and is also expressed in a subset of $Foxp3^+$ Treg cells associated with enhanced function and tissue residency (Hoyler et al., 2012; Mjösberg et al., 2012; Wohlfert et al., 2011; Zheng and Flavell, 1997). We found that lung $GATA3^+$ Treg cells were also strongly and preferentially induced by papain and IL-33, compared to $GATA3^-$ Treg cells (17.7-fold compared to 7.1-fold increase, respectively) (Figures S1A–S1D). $GATA3^+$ Treg cells in control (PBS), papain-, or IL-33-exposed lungs were likely thymus derived, as indicated by co-expression of the transcription factor Helios and the vascular endothelial growth factor (VEGF) co-receptor neuropillin (Nrp)-1 (Figures 1A, 1B, and S1E; Thornton et al., 2010; Yadav et al., 2012). $GATA3^+$ Treg cells also expressed more CTLA4 compared to $GATA3^-$ Treg cells in naive and IL-33-treated mice (Figures 1C and S1F). We purified Th2 cells, $GATA3^+$ and $GATA3^-$ Treg cells, and ILC2s from naive and IL-33-treated $Foxp3^{EGFP\text{-}DTR/+}Gata3^{hCD2/+}$ mice and performed RNA-seq gene expression analysis (Figures 1D, S1G, and S1H, and Tables S1 and S2). Gene expression data were consistent with flow cytometry findings. Moreover, we observed substantial overlap between ILC2s, $GATA3^+$ Treg cells, and Th2 cells during homeostasis and after *in vivo* IL-33 stimulation, supporting the idea of shared regulatory and functional programs between these cells (Panduro et al., 2016; Siede et al., 2016).

IL-33 is known to have direct effects on Treg cells, which express its receptor ST2 (Figure S1I; Schiering et al., 2014). However, we observed no significant proliferative advantage of $ST2^+$ over $ST2^-$ Treg cells after IL-33-induced expansion as assessed by Ki67 staining, or in an adoptive transfer experiment of mixed WT and $Il1rl1^{-/-}$ $CD4^+$ T cells, thus suggesting that an alternative mechanism contributes to IL-33-mediated Treg cell expansion (Figures S1J–S1M). Given that ILC2s can regulate Th2 cells *in vivo* via direct (Drake et al., 2014; Mirchandani et al., 2014; Oliphant et al., 2014) and indirect (Halim et al., 2014, 2016) mechanisms and can regulate Treg cells via an ICOS-ICOSL interaction *in vitro* (Molofsky et al., 2015), we tested whether IL-33-driven Treg cell expansion was ILC2 dependent in two models of ILC2 deficiency (Figures S2A and S2F). The 2W1S peptide immunogen, co-administered with IL-33, was used to track antigen-specific $CD4^+$ T cell responses using the 2W1S:I-A^b MHCII tetramer (Moon et al., 2007). We found that both antigen-specific and non-specific (2W1S:$tetramer^{+\text{ and }-}$) Treg and Th2 cell responses were impaired in ILC2-deficient $Il7ra^{Cre/+}Rora^{fl/fl}$ mice (Figures 1E, S2B, and S2C). As a subset of $CD4^+$ T cells can also express *Rora*, we generated

(F) Mice were treated with IL-33 (i.n., day 0 and 1) followed by quantification of $Foxp3^+GATA3^{+\text{ and }-}$ Treg cells and $Foxp3^-GATA3^+$ Th2 cells in the lung on day 5.
(G) PBS- or diphtheria toxin (DTX)-treated ICOS-T mice were administered with IL-33 and 2W1S-peptide as indicated (i.n., day 0 and 1), followed by quantification of 2W1S-$Tetramer^{+/-}$ $Foxp3^+GATA3^+$ Treg and $Foxp3^-GATA3^+$ Th2 cells in the mLN on day 5.
Bar graphs indicate mean (±SEM). (A)–(C), three repeat experiments, mean percent gated population in (A); (D), single experiment; (E), ANOVA, three repeat experiments; (F), ANOVA, two repeat experiments; (G), ANOVA, three repeat experiments. ns = not significant, *$p \leq 0.05$, **$p \leq 0.01$, ***$p \leq 0.001$, ****$p \leq 0.0001$. See also Figures S1 and S2.

Cd4$^{Cre/+}$*Rora*$^{fl/fl}$ mice, which showed no defect in type 2 immune-mediated expulsion of *N. brasiliensis*, or in Th2 or Treg cell numbers after allergen or IL-33 challenge, supporting our previous *in vitro* results (Figures 1F, S2D, and S2E; Halim et al., 2012b; Wong et al., 2012). Additionally, using a separate model of ILC2 deletion in diphtheria toxin (DTx)-treated ICOS-T mice (Oliphant et al., 2014), we observed a similar defect in GATA3^{+} Treg and Th2 cell responses after IL-33 + 2W1S peptide administration (Figures 1G and S2F–S2H). As reported previously, ILC3s may also be impaired by DTx in *Icos*$^{fl\text{-}DTR\text{-}fl/+}$ *Cd4*$^{Cre/+}$ (ICOS-T) mice, although lung ILC3s are sparse and not substantially affected by IL-33-driven inflammation (Figures S2G and S2H). Further, to investigate a potential role for B cells, we injected B cell-deficient *Ighm*$^{-/-}$ (μMT) mice with IL-33, followed by analysis for Th2 and Treg cells. We observed no significant difference in IL-33-driven Treg and Th2 cell expansion in the lungs of μMT compared to WT mice (Figures S2I and S2J). Moreover, immunofluorescence microscopy analysis of IL-33-treated WT mice showed co-localization of Treg cells and ILC2s in the lungs (Figure S2K), as reported previously (Molofsky et al., 2015). Thus, ILC2s are critical for allergen- and IL-33-induced expansion of Th2 cells and GATA3^{-} and GATA3^{+} Treg cells.

OX40 Is Required for Expansion of Adaptive Type 2 Immunity by IL-33

We investigated possible cell-to-cell interactions that may influence IL-33-driven Th2 and Treg cell expansion and first focused on T cell co-receptors. Gene expression analysis for the tumor necrosis factor (TNF) receptor-superfamily was conducted using lung ILC2s and other IMMGEN (Heng et al., 2008) immune cell datasets (Figure 2A). We observed high expression of *Tnfrsf4* (OX40), an important T cell co-stimulatory receptor (Croft, 2010), on Treg and activated CD4^{+} T cells. We confirmed that OX40 was expressed on lung GATA3^{+} and GATA3^{-} Treg cells in PBS, papain-, or IL-33-stimulated mice, but not Foxp3^{-}GATA3^{-}CD4^{+} T cells (Figures 2B, 2C, and S3A). OX40 was also expressed on a smaller proportion of Th2 cells after IL-33 administration (Figure 2B). We hypothesized that OX40 may be important for mediating the IL-33-driven expansion of Th2 and Treg cells. Using *Tnfrsf4*$^{-/-}$ mice, we determined that the OX40 co-stimulatory molecule was essential for IL-33-mediated Treg cell expansion in the lungs (Figures 2D–2F). We observed similar requirements of OX40 for the development of lung Th2 cells in response to IL-33, while ILC2 expansion was unaffected (Figure S3B). *Tnfrsf4*$^{-/-}$ mice also showed a similar defect in adaptive type 2 immunity after intranasal administration of papain (Figures 2G–2I). Thus, OX40 signaling is critical for the induction of IL-33-dependent adaptive type 2 immunity.

ILC2s Selectively Express OX40L in Response to IL-33

Next, we identified the critical OX40L-expressing cells by analyzing gene expression for *Tnfsf4* and other TNF superfamily ligand transcripts. We found that *Tnfsf4* (OX40L) gene expression was highest in naive lung ILC2s and substantially lower in any of the other tested immune cells (Figure 3A). To confirm these findings, we assessed OX40L expression on lung immune cells in mice stimulated intranasally with IL-25, IL-33, TSLP, papain, LPS, or PBS. While control mice did not show OX40L protein expression on any cells that we analyzed (ILC2s, B cells, CD4^{+} and CD8^{+} T cells, DCs, Macs, and NK cells), only ILC2s showed a significant induction of OX40L surface expression upon IL-33, papain, and to a lesser degree IL-25, stimulation (Figures 3B, 3C, and S4A–S4C). Moreover, lung DCs from adult mice failed to induce OX40L expression in response to intranasal administration of any of these stimuli, including two separate TSLP formulations and anti-CD40 mAb treatment (Figure S4D). Also, OX40L^{+} ILC2s significantly outnumbered OX40L^{+} DCs after IL-33 administration and also exhibited higher intensity surface staining of OX40L (Figure S4E). Notably, human ILC2s also strongly induced OX40L after IL-33 stimulation (Figure S4F). Additionally, IL-33 administration to mice by intranasal (i.n.) or intraperitoneal (i.p.) routes rapidly (day 2) increased Treg cell proliferation in the lung but not mediastinal lymph node (mLN), while ILC2s at both sites were activated and expressed ST2 (Figures 3D and S4G). When we measured the expression of OX40L on ILC2s after IL-33 administration, we observed that lung ILC2s expressed OX40L, while mLN ILC2s and ILC3s did not (Figure 3E). Lastly, proliferation of total CD4^{+} T cells was not significantly affected in either lung or mLN by IL-33 administration (Figure S4H). This led us to speculate that IL-33-driven local expansion of adaptive type 2 immune cells is governed by ILC2-restricted OX40L expression.

OX40L Expression by ILC2s Correlates with Tissue-Specific T Cell Proliferation

To further ascertain the importance of OX40L expression by ILC2s following IL-33-driven induction of adaptive type 2 immunity, we crossed *Tnfsf4*$^{fl/fl}$ mice with *Il7r*$^{Cre/+}$ mice. Although other immune cells such as ILC3s and CD4^{+} T cells also express IL7Rα, we did not observe OX40L expression in these populations. Efficient deletion of OX40L on ILC2s was achieved in *Il7r*$^{Cre/+}$*Tnfsf4*$^{fl/fl}$ mice, as indicated by reduced expression of OX40L on lung ILC2s after IL-33 administration (Figures 4A and S5A). Next, we asked whether ILC2-targeted deletion of OX40L influenced the IL-33-driven effects on adaptive type 2 immunity. We administered IL-33 to *Il7r*$^{Cre/+}$, *Il7r*$^{Cre/+}$*Rora*$^{fl/fl}$, and *Il7r*$^{Cre/+}$*Tnfsf4*$^{fl/fl}$ mice on days 0 and 1, followed by analysis on day 5. Notably, *Il7r*$^{Cre/+}$*Tnfsf4*$^{fl/fl}$ mice failed to induce Th2 cells and GATA3^{+} and GATA3^{-} Treg cell responses in the lungs after treatment (Figures 4B–4D). The observed defect produced a phenotype similar to naive control or IL-33-treated *Il7r*$^{Cre/+}$*Rora*$^{fl/fl}$ mice. We subsequently investigated lung, large intestine, and perigonadal adipose tissue for the IL-33/ILC2/OX40L-driven effect on adaptive type 2 immunity. Here we noted clearly divergent phenotypes with OX40L expression induced only on lung and adipose tissue-resident ILC2s but not in the intestine (Figure 4E). Nevertheless, ILC2 and ILC3 numbers in *Il7r*$^{Cre/+}$*Tnfsf4*$^{fl/fl}$ mice were similar to those in control mice (Figures 4F, S5B, and S5D). Moreover, only these two sites exhibited IL-33-driven expansion of Th2 cells and GATA3^{+} and GATA3^{-} Treg cells (Figures 4G and S5C). Importantly, ILC2-targeted deletion of OX40L phenocopied ILC2 deficiency. This indicated the critical nature of OX40L expression by ILC2s for establishing tissue-specific adaptive type 2 immunity.

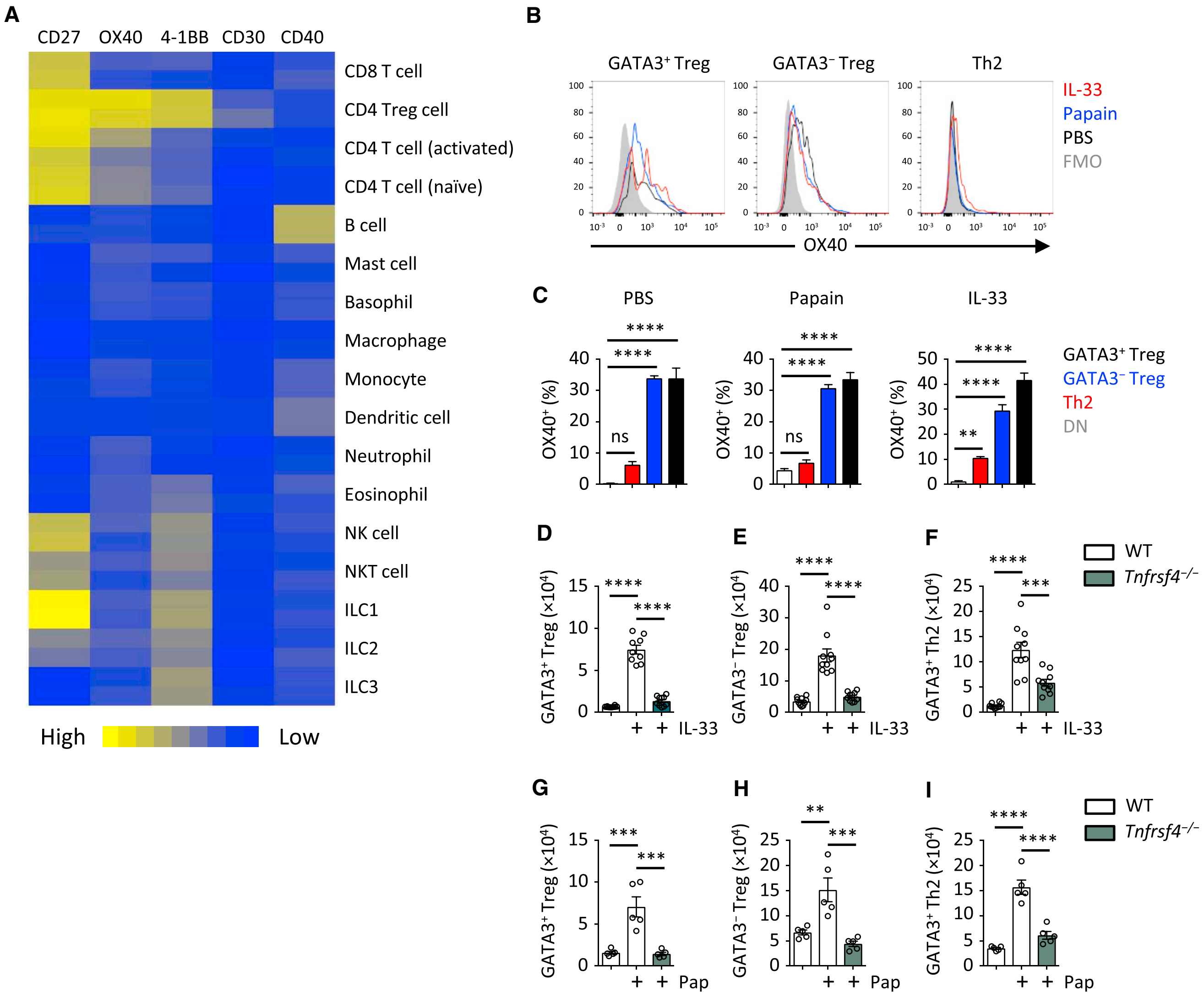

Figure 2. OX40 Is Critical for Th2 and Treg Cell Response to IL-33 and Papain

(A) TNFRSF gene expression was analyzed in the indicated immune cell populations using IMMGEN and naive lung ILC2 microarray data.

(B and C) OX40 expression was analyzed on lung GATA3$^+$ and GATA3$^-$ Treg and Th2 cells on day 5 from WT mice treated with PBS, papain, or IL-33 (i.n., day 0 and 1). Fluorescence minus one (FMO) control was used to establish gating threshold. Percent OX40$^+$ cells were calculated in the indicated populations and treatments and compared to Foxp3$^-$GATA3$^-$ (DN) CD4$^+$ T cells.

(D–F) Lung GATA3$^+$ and GATA3$^-$ Treg and Th2 cells were quantified from WT and *Tnfrsf4*$^{-/-}$ mice on day 5 after IL-33 treatment (i.n., day 0 and 1).

(G–I) Lung GATA3$^+$ and GATA3$^-$ Treg and Th2 cells were quantified from WT and *Tnfrsf4*$^{-/-}$ mice on day 5 after papain (Pap) treatment (i.n., day 0 and 1).

Bar graphs indicate mean (±SEM). (A), two independent datasets per group; (B) and (C), ANOVA, two repeat experiments; (D)–(F), ANOVA, two repeat experiments; (G)–(I), ANOVA, two repeat experiments. ***p ≤ 0.001, ****p ≤ 0.0001. See also Figure S3.

OX40L on ILC2s Is Specifically Required for IL-33-Driven Effects on Adaptive Type 2 Immunity

OX40L is reported on other IL7Rα$^+$ cell types, including CD4$^+$ T cells and RORγt$^+$ ILC3s, and is widely described on CD11c$^+$ DCs (Kim et al., 2005; Webb et al., 2016). While we observed only OX40L on ILC2s after IL-33 stimulation, IL7Rα^{Cre}-driven deletion of OX40L is not entirely ILC2 specific. To better control for possible OX40L signaling contributions from other immune cells, we intercrossed *Tnfsf4*$^{fl/fl}$ mice with *Itgax*^{Cre}, *Rorc*$^{Cre/+}$, and *Cd4*$^{Cre/+}$ mice to further exclude non-ILC2-driven effects. As before, we administered IL-33 to the indicated control or OX40L-targeted mice, followed by analysis for induction of adaptive type 2 immunity. While ILC2-deficient and ILC2-targeted OX40L-deficient *Il7r*$^{Cre/+}$*Tnfsf4*$^{fl/fl}$ mice both failed to induce GATA3$^+$ Treg, GATA3$^-$ Treg, and Th2 cells, mice with DC-targeted (*Itgax*^{Cre}*Tnfsf4*$^{fl/fl}$), ILC3-targeted (*Rorc*$^{Cre/+}$ *Tnfsf4*$^{fl/fl}$), and CD4$^+$ T cell-targeted (*Cd4*$^{Cre/+}$*Tnfsf4*$^{fl/fl}$) OX40L deletion showed no deficit compared to OX40L-sufficient control mice (Figures 5A–5C). Finally, we generated *Il7r*$^{Cre/+}$*Rora*$^{fl/fl}$ + *Il7r*$^{Cre/+}$*Tnfsf4*$^{fl/fl}$ mixed bone marrow (BM) chimeric mice, in which ILC2s are OX40L deficient, to further investigate the role of OX40L on ILC2s (Figure S6D). The mixed BM chimeras

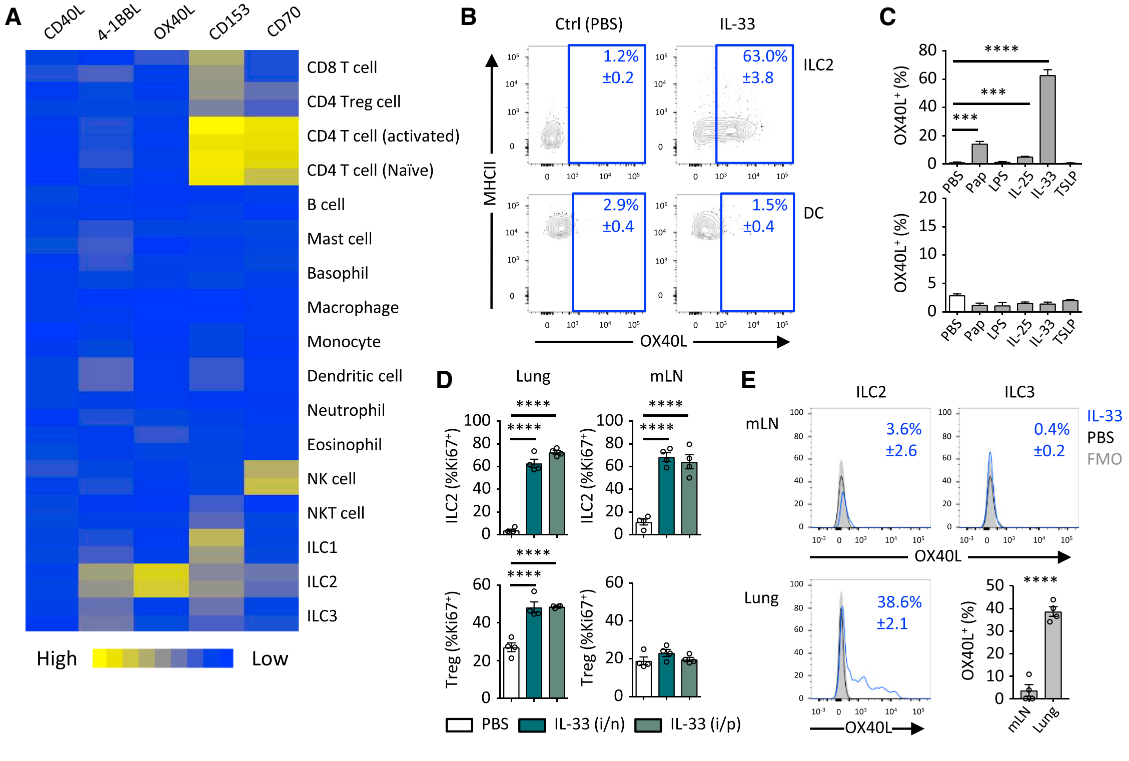

Figure 3. ILC2s Are the Main OX40L-Expressing Cell in Response to IL-33

(A) TNFSF gene expression was analyzed in the indicated immune cell populations using IMMGEN and naive lung ILC2 microarray data.

(B) OX40L expression was measured on lung DCs or ILC2s in WT mice on day 2 after treatment with PBS or IL-33 (i.n., day 0 and 1).

(C) The percentage of OX40L$^+$ ILC2s (top) and DCs (bottom) was measured on day 2 in response to the indicated stimuli (i.n., day 0 and 1).

(D) The percent of Ki67$^+$ ILC2s and Treg cells was measured on day 2 after treatment with PBS or IL-33 (day 0 and 1).

(E) OX40L expression was measured on ILC2s and ILC3s in the lung or mLN on day 2 after PBS or IL-33 treatment (i.n., day 0 and 1).

Numbers in (B) and (E) indicate percent (mean ± SD) gated populations. (A), two independent datasets per group; (B), three repeat experiments; (C), ANOVA, two repeat experiments; (D), ANOVA, two repeat experiments; (E), two-tailed Student's t test, two repeat experiments. ***$p \leq 0.001$, ****$p \leq 0.0001$. See also Figure S4.

showed a significant reduction, similar to ILC2-deficient BM chimeric mice, in GATA3$^+$ and GATA3$^-$ Treg cells after IL-33 administration compared to control mice (Figures 5D and 5E). Thus, OX40L expression on ILC2s is essential for IL-33-driven activation of adaptive type 2 immunity.

IL-33-ILC2-OX40L Pathway Is Critical for Allergen-Induced Adaptive Type 2 Immunity

Protease allergens are potent inducers of IL-33 in the airways, which is a central mechanism by which they drive innate and adaptive type 2 immunity (Halim et al., 2012a; Hammad and Lambrecht, 2015). In addition to Th2 cells, we investigated the role of IL-33 in mediating allergen-induced Treg cell responses in the lungs. Administration of the protease allergen papain led to an induction of lung GATA3$^+$ and GATA3$^-$ Treg cells on day 5 in WT mice (Figures 6A, 6B, and S7A). Papain-induced Treg cell responses were significantly reduced in *Il33*$^{cit/cit}$ (*Il33*$^{-/-}$) or *Il1rl1*$^{-/-}$ mice (Figures 6A and 6B), indicating the importance of IL-33 for mediating allergen-induced expansion of Treg cells in the airways. As expected, Th2 cells were also significantly reduced in *Il33*$^{cit/cit}$ or *Il1rl1*$^{-/-}$ mice compared to WT controls (Figures 6C and 6D). We then investigated the role of ILC2-expressed OX40L for driving adaptive type 2 immunity in the lungs after papain exposure. We observed a failure of Th2 cells and GATA3$^+$ and GATA3$^-$ Treg cells to develop in the lungs of *Il7r*$^{Cre/+}$*Tnfsf4*$^{fl/fl}$ mice (Figures 6A–6D). Next we exposed *Il7r*$^{Cre/+}$, *Il7r*$^{Cre/+}$*Rora*$^{fl/fl}$, and *Il7r*$^{Cre/+}$*Tnfsf4*$^{fl/fl}$ mice to repeat exposures of papain after initial sensitization, followed by analysis of the lungs for multiple parameters of innate and adaptive type 2 inflammation (Figure S7B). On day 24 we found that *Il7r*$^{Cre/+}$*Tnfsf4*$^{fl/fl}$ mice had significant reductions in Th2 cells and GATA3$^+$ and GATA3$^-$ Treg cells compared to *Il7r*$^{Cre/+}$ controls (Figures S7C–S7E). These reductions were similar to those observed in ILC2-deficient mice. We also observed significant reductions in lung eosinophilia (Figure 6E) and M2 polarization by lung macrophages as assessed by intracellular staining for RELMα (Figures 6F and 6G; Nair et al., 2009). We further investigated whether OX40L expression by ILC2s may influence adaptive type 2 immunity to another allergen. Administration of *Alternaria alternata* extract promoted an increase in lung Th2 cells, but also GATA3$^{+\ and\ -}$ Treg cells by day 9, that was impaired in *Il7r*$^{Cre/+}$*Tnfsf4*$^{fl/fl}$ mice (Figure 6H). Moreover, we found that the *A. alternata* induced increase in serum IgE was reduced in *Il7r*$^{Cre/+}$*Tnfsf4*$^{fl/fl}$ mice (Figure 6I). Interestingly, CD11c^{Cre} targeted OX40L deletion also resulted in reduced Th2 and Treg cells after *A. alternate*-induced airway inflammation (Figure S7F),

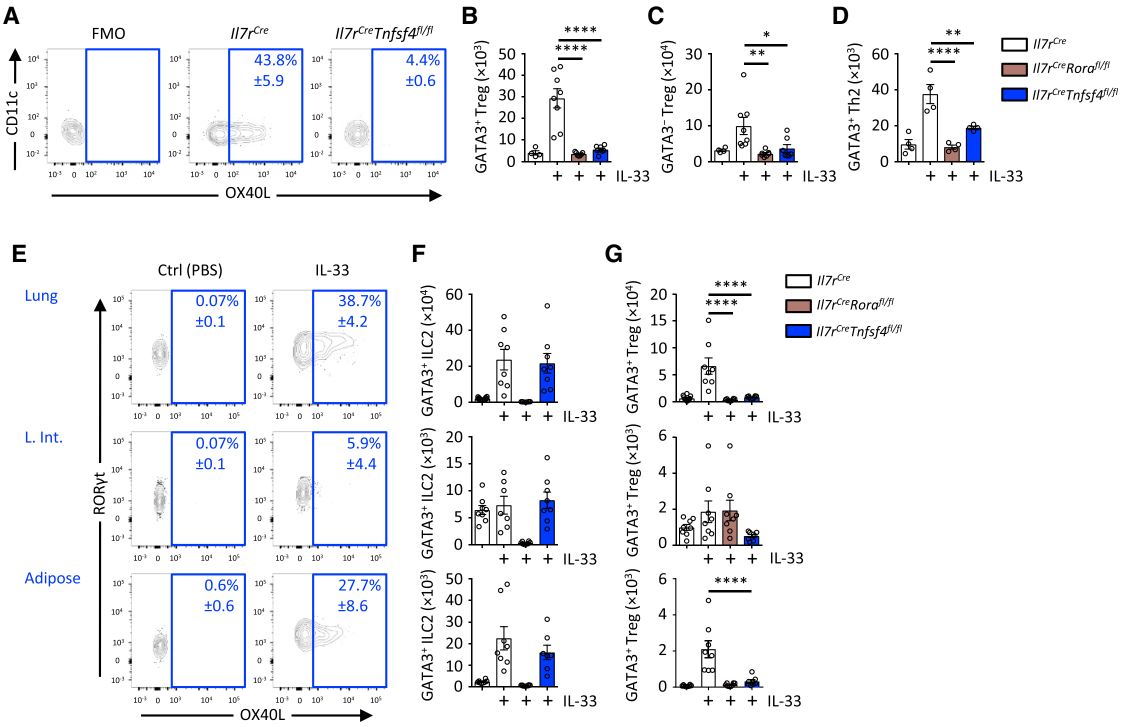

Figure 4. OX40L Expression by ILC2s Is Essential for Tissue-Specific Adaptive Immune Response to IL-33

(A) OX40L expression on lung ILC2s in the specified genotypes on day 2 after IL-33 administration (i.n., day 0 and 1).

(B–D) Lung GATA3$^+$ and GATA3$^-$ Treg and Th2 cell numbers were quantified on day 5 after treatment with PBS or IL-33 (i.n., days 0 and 1).

(E–G) OX40L expression on ILC2s was measured in the indicated tissues (L. int., large intestine) after treatment with PBS or IL-33 (days 0 and 1) on day 2 (E), while the number of ILC2s (F) and GATA3$^+$ Treg cells was quantified on day 5 (G).

Numbers in (A) and (E) indicate percent (mean ± SD) gated populations. Bar graphs indicate mean (±SEM). (A), three repeat experiments; (B)–(D), ANOVA, two repeat experiments; (E)–(G), ANOVA, two repeat experiments. *$p \leq 0.05$, **$p \leq 0.01$, ****$p \leq 0.0001$. See also Figure S5.

suggesting that both DC- and ILC2-derived OX40L contribute to the response. Thus, ILC2-expressed OX40L is critical for mediating ILC2-induced innate and adaptive type 2 inflammation to allergen exposure in an IL-33-dependent pathway.

OX40L-Expressing ILC2s Are Essential for Adaptive Type 2 Immunity to Helminth Challenge

To interrogate the importance of the ILC2-OX40L axis in complex immune challenges, we used the prototypical type 2 immunity-inducing parasitic helminth, *N. brasiliensis*, which traffics through the lung after infection, inducing profound and prolonged type 2 inflammation (Mohrs et al., 2001). Besides Th2 cells, Treg cells are known to play an important role for wound healing and tissue remodeling of the lung (Chen et al., 2012; Taylor et al., 2012). First, we characterized the adaptive type 2 immune response in the lungs of mice on day 28 after *N. brasiliensis* infection and observed substantial increases in GATA3$^+$ and GATA3$^-$ Treg cells and Th2 cells in control mice (Figures 7A–7C and S7G). By contrast, *Il7r*$^{Cre/+}$*Tnfsf4*$^{fl/fl}$ mice exhibited a failure of the lung Th2 cells and GATA3$^+$ and GATA3$^-$ Treg cells to expand, with numbers remaining similar to uninfected control mice. Notably, this impairment mirrored that measured in *Il7r*$^{Cre/+}$*Rora*$^{fl/fl}$ mice (Figures 7A–7C). Lung histology demonstrated that *Il7r*$^{Cre/+}$*Tnfsf4*$^{fl/fl}$ mice had reduced histological features of inflammation compared to infected control mice (Figure 7D), again similar to those in *Il7r*$^{Cre/+}$*Rora*$^{fl/fl}$ mice. While infected control mice mounted a prolific eosinophil response, we observed an impairment in *Il7r*$^{Cre/+}$*Tnfsf4*$^{fl/fl}$ mice to induce eosinophilia in the lung (Figure 7E) or BAL (Figure 7F). Similar impairments were observed when we quantified RELMα^+ M2 polarized lung macrophages (Figure 7G) and BAL concentrations of IL-4 and IL-5 (Figure 7H), while IL-13$^+$ Th2 cells were also significantly reduced in the lungs of *Il7r*$^{Cre/+}$*Tnfsf4*$^{fl/fl}$ compared to control mice (Figure 7I). Moreover, by day 28 after infection, we observed a reduction of adaptive type 2 CD4$^+$ T cells in the mLN, and total lung IgE concentration of both infected *Il7r*$^{Cre/+}$*Rora*$^{fl/fl}$ and *Il7r*$^{Cre/+}$*Tnfsf4*$^{fl/fl}$ mice compared to controls (Figures 7J–7M). Furthermore, at 5 days after *N. brasiliensis* infection, we observed a modest increase in worm numbers of *Il7r*$^{Cre/+}$*Tnfsf4*$^{fl/fl}$ mice compared to control mice (Figure 7N). Of note, while absolute cell numbers were reduced, we observed no reduction in Th2 cell frequency in infected *Il7r*$^{Cre/+}$*Rora*$^{fl/fl}$ or *Il7r*$^{Cre/+}$*Tnfsf4*$^{fl/fl}$ mice compared to control mice. Moreover, no significant differences were observed in type 1 immunity (IFN-γ production), NK cells, and CD8$^+$ T cells of both *Il7r*$^{Cre/+}$*Rora*$^{fl/fl}$ and *Il7r*$^{Cre/+}$*Tnfsf4*$^{fl/fl}$ mice compared to controls after infection (Figures S7H–S7K). Thus, these results indicate that the IL-33/ILC2/OX40L axis is broadly important for orchestrating innate and adaptive type 2 immunity following helminth challenge.

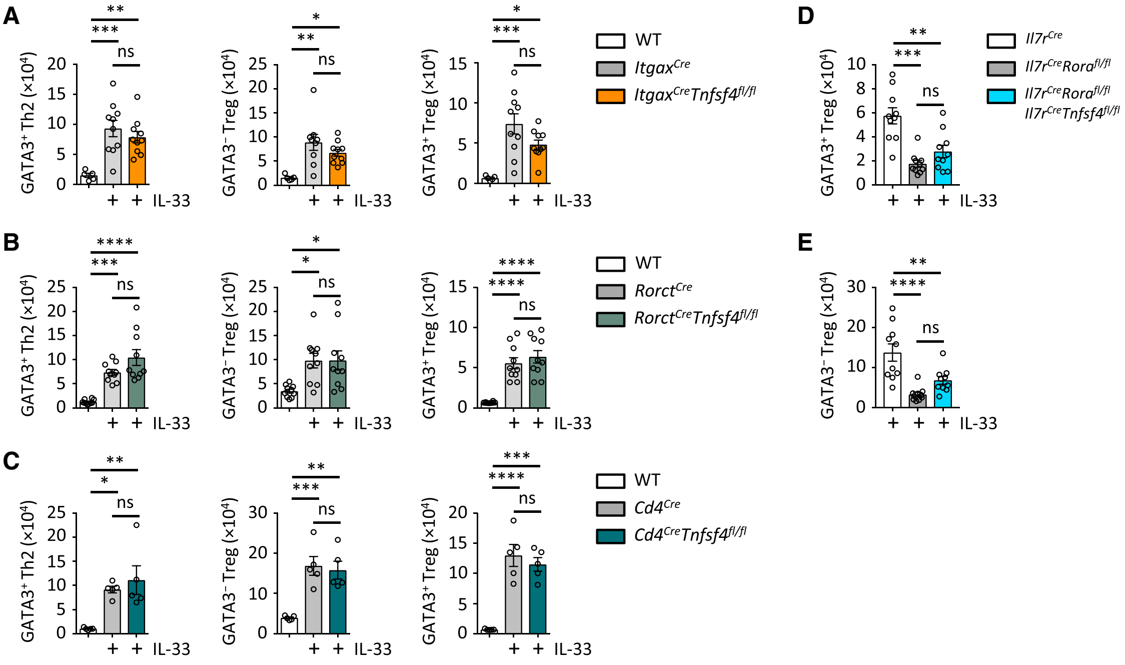

Figure 5. OX40L-Driven Response to IL-33 Is Restricted to ILC2s

(A–C) Lung GATA3^{+} and GATA3^{-} Treg cells and Th2 cells were quantified on day 5 in the specified genotypes, treated with PBS or IL-33 (i.n., days 0 and 1).

(D and E) Bone marrow and mixed-bone marrow chimeric mice were created with the indicated genotypes. 6 to 7 months after bone marrow transfer, mice received IL-33 (i.n., days 0 and 1) followed by quantification of lung GATA3^{+} Treg (D) and GATA3^{-} Treg (E) cells on day 5.

Bar graphs indicate mean (±SEM). ANOVA, two repeat experiments. ns = not significant, *p ≤ 0.05, **p ≤ 0.01, ***p ≤ 0.001, ***p ≤ 0.0001. See also Figure S6.

DISCUSSION

The potential role of ILC2s as key potentiators of immune activation, performing as intermediaries between the damaged epithelium and the adaptive immune system, is currently an important and unresolved question. While previous studies have started to reveal mechanisms by which ILC2s can influence Th2 cell priming (Halim et al., 2014, 2016; Mirchandani et al., 2014; Oliphant et al., 2014), the continued importance of ILC2s in the presence of adaptive type 2 immunity is unclear (Van Dyken et al., 2016; Vély et al., 2016), especially because Th2 and Treg cells may also be directly activated by IL-33 (Guo et al., 2015; Schiering et al., 2014).

Our results using ILC2-deficient mouse models indicated that IL-33-driven expansion of Th2 and Treg cells was dependent on ILC2s. As RORα is also expressed by T cells, it has been suggested that deletion of *Rora* in lymphocytes may directly alter T cell responses. Significantly, using *Cd4*$^{Cre/+}$*Rora*$^{fl/fl}$ mice, we excluded the possibility that *Rora* deletion results in CD4^{+} T cell-intrinsic effects that diminish adaptive immunity. Indeed, we demonstrated that mice whose T cells lack RORα mounted equivalent type 2 adaptive immune responses to parasitic worm infection, papain allergen, and IL-33 as RORα-sufficient T cell controls.

To further elucidate the mechanism by which ILC2s can orchestrate Th2 and Treg cell responses, we identified costimulatory molecules that were counter-expressed on ILC2s and T cells. Gene expression data highlighted the potential importance of OX40/OX40L interactions, which have been linked previously with the development of Th2 cell responses (Croft, 2010; Webb et al., 2016). OX40 is expressed on memory T cells after TCR-mediated recall responses (Croft, 2010) and is also associated with Treg cell homeostasis and function (Takeda et al., 2004). Additionally, we discovered that OX40L, which has been reported on other immune cells in response to type 2 alarmins (Croft, 2010; Webb et al., 2016), was increased on lung ILC2s in response to IL-33.

Notably, we were unable to detect OX40L on lung-resident dendritic cells, or other lung cell populations, even after treating mice with TSLP. Although OX40L mRNA expression is reported in many models of type 2 inflammation, and OX40L protein expression can be induced on *in vitro* cell-cultured mouse and human DCs stimulated with various cytokines (e.g., TSLP) and antigens, the cellular expression of OX40L protein *in vivo* remains relatively opaque. Nevertheless, while alarmin stimulation did not induce OX40L expression on lung-resident DCs, our study with *A. alternata*-induced lung inflammation clearly showed a role for OX40L expression by CD11c^{+} cells, confirming previous findings of impaired Th2 cell priming of OX40L-deficient DCs (Jenkins et al., 2007). As we did not observe OX40L on ILC2s in the draining mLNs, it appears that separate functions of OX40L are enforced by distinct cell types.

Moreover, while IL-33-mediated expansion of Th2 and Treg cells may involve an antigen-independent mechanism solely reliant on ILC2s, more complex antigens such as *A. alternata* and house dust mite activate multiple cellular targets, including DCs (de Kleer et al., 2016). Similarly, neonatal mouse DCs in the lung transiently express OX40L, suggesting differential

CellPress

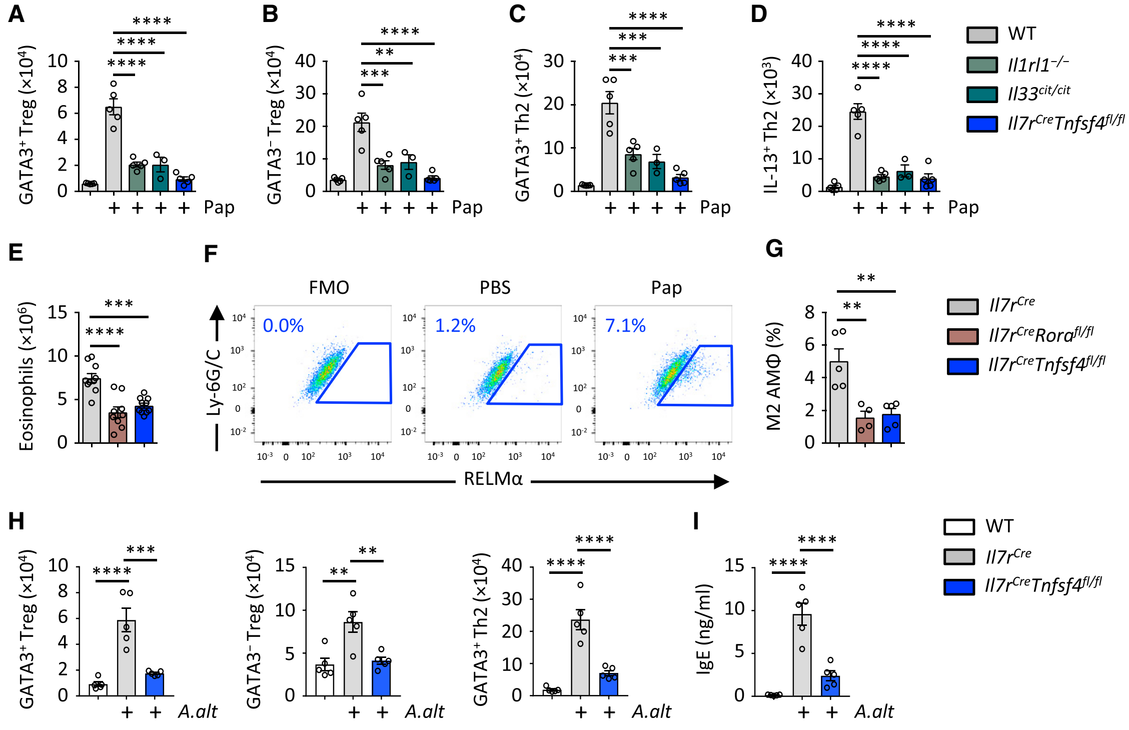

Figure 6. OX40L on ILC2s Orchestrates Adaptive Type 2 Immunity to Allergens

(A–D) Mice of the specified genotypes were treated with PBS or papain (Pap) (i.n., day 0 and 1), followed by quantification of lung Th2, GATA3$^+$ Treg, and GATA3$^-$ Treg cells on day 5 (A–C). Whole lung cell suspensions were re-stimulated with PMA and ionomycin, followed by quantification of IL-13$^+$ Th2 cells by intracellular staining (D).

(E–G) Mice were treated with papain on days 0, 1, 14, and 21, followed by quantification on day 24 of lung eosinophils (E), and detection (F) and quantification (G) of RELMα^+ M2 alveolar (CD45$^+$SiglecF$^+$CD11c$^+$CD11b$^-$F4/80$^+$) macrophages (MΦ).

(H and I) Mice were treated with *A. alternata* (*A.alt*) (i.n., days 0 and 1), followed by quantification on day 9 of lung Th2, GATA3$^+$ Treg, and GATA3$^-$ Treg cells (H) and serum IgE concentration (I).

Bar graphs indicate mean (±SEM). (A)–(D), ANOVA, two repeat experiments; (E), ANOVA, three repeat experiments; (F) and (G), ANOVA, two repeat experiments, representative gate shown in (F); (H) and (I), ANOVA, two repeat experiments. **p $\leq$ 0.01, ***p $\leq$ 0.001, ****p $\leq$ 0.0001. See also Figure S7.

expression patterns dependent on developmental stage (de Kleer et al., 2016). Thus, ILC2- and DC-expressed OX40L may play partially compensatory roles and further study is required to resolve the complexity of how differing temporal expression, tissue environment, and the nature of the antigen encountered may influence the induction of these pathways in protecting the host.

We observed that OX40-deficient mice and mice with a conditional deletion of OX40L (*Il7r*$^{Cre/+}$*Tnfsf4*$^{fl/fl}$) had significantly impaired Th2 and Treg cell responses following IL-33 administration, a phenotype similar to ILC2-deficient mice. Furthermore, mixed BM chimeric mice, in which only ILC2s were OX40L deficient, also failed to induce Treg cells following IL-33 administration. By contrast, conditional deletion of OX40L from T cells, dendritic cells, and ILC3s resulted in normal IL-33-induced responses. The deletion of OX40L from ILC2s also curtailed the onset of type 2 immunity in the lungs of mice administered papain allergen or infected with parasitic helminths. Thus, OX40L expression on ILC2s, but not DCs, is essential for the robust onset of IL-33-induced type 2-mediated lung inflammation.

Previous studies have demonstrated that OX40- and OX40L-deficient mice fail to develop type 2 responses characteristic of allergic lung inflammation (Hoshino et al., 2003; Jember et al., 2001) and showed that OX40L-deficient (*Tnfsf4*$^{-/-}$) mice were less efficient at responding to *Heligmosomoides polygyrus* (Ekkens et al., 2003). We now establish ILC2s, through their tissue-specific expression of OX40L in response to IL-33, as critical players in the local expansion of OX40-expressing Th2 and Treg cells and the initiation and maintenance of robust type 2 responses in the lung. The co-induction of Th2 and Treg cells via the same signaling pathways may serve to limit allergic inflammation, as indicated by the suppressive role of Treg cells on Th2 cell-driven lung inflammation (Suto et al., 2001), while also promoting wound healing such as in response to helminth-induced damage (Pulendran and Artis, 2012). Indeed, we showed that *N. brasiliensis*-induced adaptive type 2 immunity, important for lung damage repair (Chen et al., 2012), is dependent on OX40L expression on ILC2s. Accordingly, we observed a profound reduction in eosinophils, alternatively activated Macs, and type 2 effector cytokines. Importantly, ILC2s and Treg and Th2 cells were detectable in normal numbers at baseline in *Il7r*$^{Cre/+}$*Tnfsf4*$^{fl/fl}$ mice, and type 1 immunity was unaffected after immune challenge.

OX40L can be regulated in an age- and tissue-specific manner and can be induced on a range of immune cells under various

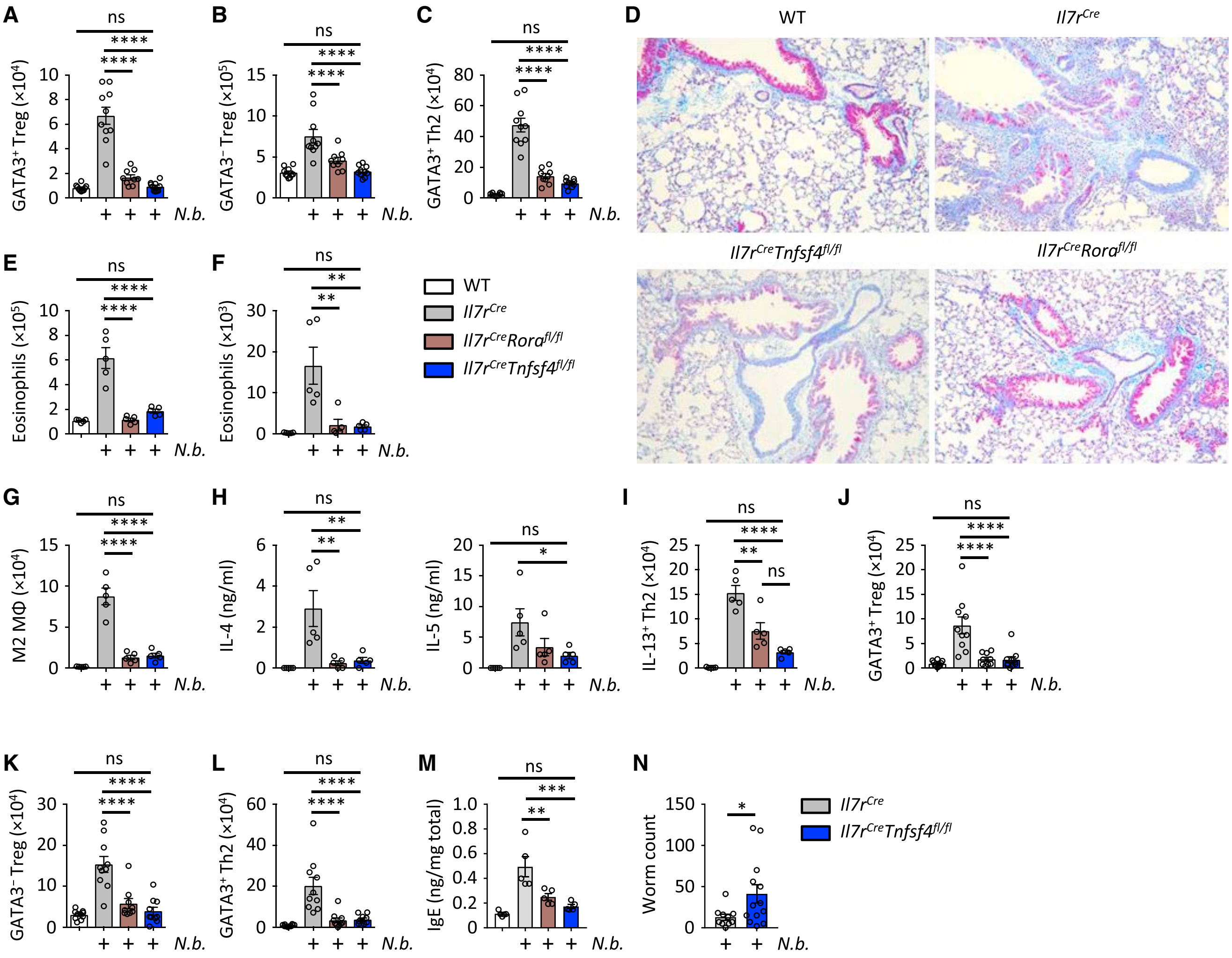

Figure 7. ILC2-Expressed OX40L Is Essential for Airway Adaptive Type 2 Immune Response to Helminth Infection

Mice of the specified genotypes were infected with *Nippostrongylus brasiliensis* (*N.b.*) on day 0, followed by analysis on day 28 (or day 5) of:

(A–C) Lung Th2, GATA3$^+$ Treg, and GATA3$^-$ Treg cell numbers.

(D) Representative lung histology (Mason's trichrome).

(E and F) Lung (E) and bronchoalveolar lavage (F) eosinophil numbers.

(G) Lung RELMα^+ M2 macrophage (MΦ) numbers.

(H) Bronchoalveolar lavage IL-4 and IL-5 cytokine concentrations.

(I) Whole lung cell suspensions were re-stimulated with PMA and ionomycin, followed by quantification of lung IL-13$^+$ Th2 cell numbers by intracellular staining.

(J–L) Mediastinal lymph node Th2, GATA3$^+$ Treg, and GATA3$^-$ Treg cell numbers.

(M) Concentration of IgE present in lung homogenate, normalized for total protein content.

(N) Intestinal worm burden of indicated mouse genotypes 5 days post infection.

Bar graphs indicate mean (±SEM). (A)–(C), ANOVA, three repeat experiments; (D), two repeat experiments; (E)–(I), ANOVA, two repeat experiments; (J)–(L), ANOVA, three repeat experiments; (M), ANOVA, two repeat experiments; (N), two-tailed Student's t test, two pooled experiments. ns = not significant, *$p \leq 0.05$, **$p \leq 0.01$, ***$p \leq 0.001$, ****$p \leq 0.0001$. See also Figure S7.

inflammatory conditions (Croft, 2010; de Kleer et al., 2016; Webb et al., 2016). The availability of a conditional OX40L allele will be of significant importance in continuing to identify critical cellular sources of OX40L in mouse models. For example, whether OX40L on ILC2s, in addition to DCs, is also instructive during neonatal establishment of persistent Th2 and Treg cells in response to IL-33 is unknown but of critical importance (de Kleer et al., 2016; Gollwitzer et al., 2014; Saluzzo et al., 2017; Steer et al., 2017). In adult mice, however, we implicated OX40L expression on ILC2s in diverse lung pathologies. Other cellular signaling molecules such as ICOSL and PD-1L may also influence cross-talk between ILC2s and T cells, although the magnitude of the ILC2-specific effect and broad expression of these ligands on other immune cells suggests a different role compared to OX40L (Molofsky et al., 2015; Schwartz et al., 2017). The tissue-specific OX40L expression profile of ILC2s was an intriguing finding that warrants further investigation. Environmental cues or developmental programs may influence the

response and/or sensitivity of ILC2s to IL-33. Intestinal ILC2s express ST2 but are known to be more sensitive to IL-25 than lung ILC2s. Nevertheless, mLN ILC2s were activated by IL-33 but do not express OX40L. Whether ILC2s are directly involved in regulating follicular T and B cell responses is an intriguing question, given our observation of reduced IgE concentrations in *Il7r*$^{Cre/+}$*Tnfsf4*$^{fl/fl}$ mice after allergen or helminth exposure, and the known function of OX40 in humoral immunity (Cortini et al., 2017; Gaspal et al., 2005; Jember et al., 2001; Tahiliani et al., 2017). Significantly, delineating the effect of OX40L expression by ILC2s and other immune cells in specific anatomical locations will aid the refinement of proposed immunotherapies targeting the OX40L/OX40 interaction (Gauvreau et al., 2014; Linch et al., 2015).

While important for establishing adaptive type 2 inflammation in the airways, we have not resolved the functional significance of the parallel ILC2-driven expansion of Treg cells. While Treg cells can suppress type 2 inflammation (Lewkowich et al., 2005; Morita et al., 2015; Suto et al., 2001), a more synergistic role is observed in cases of type 1 immune suppression, wound healing, and metabolism. This synergy, in addition to similarities in development, led to the hypothesis that Treg cell function can intersect with that of Th2 cells (Chapoval et al., 2010; Panduro et al., 2016). We indeed observed a substantial overlap in gene expression profiles between Th2 cells and Treg cells, but also ILC2s. Many of these genes such as *Irf4*, *Gata3*, *Nrp1*, *Areg*, and *Il1rl1* have shared functions between these lineages (Arpaia et al., 2015; Monticelli et al., 2011; Odegaard and Chawla, 2015; Siede et al., 2016; Vasanthakumar et al., 2015). Moreover, lung GATA3^{+} Treg cells were strongly induced by protease allergens and IL-33, and our data support previous findings that assign enhanced effector function to this subset (Wohlfert et al., 2011). Further studies are required to dissect the IL-33-ILC2-OX40L axis on specific Treg cell subsets in homeostasis and disease.

In conclusion, we have revealed a critical immune-regulator checkpoint for tissue-specific orchestration of adaptive type 2 immunity, whereby the potent effect of the alarmin IL-33 on adaptive immunity is contingent on its upregulation of OX40L by ILC2s.

STAR★METHODS

Detailed methods are provided in the online version of this paper and include the following:

- KEY RESOURCES TABLE
- CONTACT FOR REAGENT AND RESOURCE SHARING
- EXPERIMENTAL MODEL AND SUBJECT DETAILS
  - *In vivo* animal studies
  - Human studies
  - Primary cell culture
- METHOD DETAILS
  - Primary leukocyte preparation
  - Flow cytometry
  - *In vivo* stimulation
  - Adoptive CD4^{+} T cell transfer
  - Bone marrow transplantation
  - IF microscopy
  - Histology
  - Human ILC2 culture
  - Gene expression analysis
  - IgE measurement
  - RNA-seq
- QUANTIFICATION AND STATISTICAL ANALYSIS
  - Statistics
  - Additional details for main figures
- DATA AND SOFTWARE AVAILABILITY

SUPPLEMENTAL INFORMATION

Supplemental Information includes seven figures and two tables and can be found with this article online at https://doi.org/10.1016/j.immuni.2018.05.003.

ACKNOWLEDGMENTS

We thank Natalio Garbi for the *Itgax*$^{Luci.DTR.Cre}$ mice and Peter Lane for the *Tnfrsf4*$^{-/-}$ mice. We thank the MRC ARES and flow cytometry facilities, Jhuma Pramanik and the Sanger RSF, and the CRUK histology and genomics core facilities. This study was supported by grants from the UK Medical Research Council (U105178805) and Wellcome Trust (100963/Z/13/Z) (A.N.J.M.); Wellcome Trust (17966/Z/2008) (T.J.V.); EMBL and ERC (L.H.-V.); and Cancer Research UK and Wellcome Trust (204622/Z/16/Z) (T.Y.F.H.).

AUTHOR CONTRIBUTIONS

T.Y.F.H. designed and performed experiments and wrote the paper. B.M.J.R., J.A.W., H.E.J., B.K., M.D.K., and E.M.S. performed experiments. J.A.W. created the *Gata3*^{hCD2} mice. H.-R.R. provided the *Il7r*^{Cre} mice (Schlenner et al., 2010). M.B. and T.J.V. provided the *Tnfsf4*$^{fl/fl}$ mice. P.G.F. provided the *N.b.* reagent. L.H.-V. and S.A.T. provided *Cd4*$^{Cre/+}$*Rora*$^{fl/fl}$ mouse results. D.R.W. provided the *Rorct*$^{Cre/+}$*Tnfsf4*$^{fl/fl}$ and *Tnfrsf4*$^{-/-}$ mice and performed the IF microscopy (with Z.L.). A.N.J.M. supervised the project, designed the experiments, and wrote the paper.

DECLARATION OF INTERESTS

A.N.J.M. grant support: Janssen, MedImmune/Astrazeneca, and GSK. S.A.T. additional affiliations: EMBL-European Bioinformatics Institute, Cavendish Laboratory University of Cambridge, and Churchill College. L.H.-V. additional affiliation: EMBL-European Bioinformatics Institute.

Received: August 4, 2017
Revised: January 31, 2018
Accepted: May 10, 2018
Published: June 12, 2018

REFERENCES

Arpaia, N., Green, J.A., Moltedo, B., Arvey, A., Hemmers, S., Yuan, S., Treuting, P.M., and Rudensky, A.Y. (2015). A distinct function of regulatory T cells in tissue protection. Cell *162*, 1078–1089.

Chapoval, S., Dasgupta, P., Dorsey, N.J., and Keegan, A.D. (2010). Regulation of the T helper cell type 2 (Th2)/T regulatory cell (Treg) balance by IL-4 and STAT6. J. Leukoc. Biol. *87*, 1011–1018.

Chen, F., Liu, Z., Wu, W., Rozo, C., Bowdridge, S., Millman, A., Van Rooijen, N., Urban, J.F., Jr., Wynn, T.A., and Gause, W.C. (2012). An essential role for TH2-type responses in limiting acute tissue damage during experimental helminth infection. Nat. Med. *18*, 260–266.

Cortini, A., Ellinghaus, U., Malik, T.H., Cunninghame Graham, D.S., Botto, M., and Vyse, T.J. (2017). B cell OX40L supports T follicular helper cell development and contributes to SLE pathogenesis. Ann. Rheum. Dis. *76*, 2095–2103.

Croft, M. (2010). Control of immunity by the TNFR-related molecule OX40 (CD134). Annu. Rev. Immunol. *28*, 57–78.

de Kleer, I.M., Kool, M., de Bruijn, M.J., Willart, M., van Moorleghem, J., Schuijs, M.J., Plantinga, M., Beyaert, R., Hams, E., Fallon, P.G., et al. (2016). Perinatal activation of the interleukin-33 pathway promotes type 2 immunity in the developing lung. Immunity *45*, 1285–1298.

Drake, L.Y., Iijima, K., and Kita, H. (2014). Group 2 innate lymphoid cells and CD4+ T cells cooperate to mediate type 2 immune response in mice. Allergy *69*, 1300–1307.

Ekkens, M.J., Liu, Z., Liu, Q., Whitmire, J., Xiao, S., Foster, A., Pesce, J., VanNoy, J., Sharpe, A.H., Urban, J.F., and Gause, W.C. (2003). The role of OX40 ligand interactions in the development of the Th2 response to the gastrointestinal nematode parasite *Heligmosomoides polygyrus*. J. Immunol. *170*, 384–393.

Gaspal, F.M., Kim, M.Y., McConnell, F.M., Raykundalia, C., Bekiaris, V., and Lane, P.J. (2005). Mice deficient in OX40 and CD30 signals lack memory antibody responses because of deficient CD4 T cell memory. J. Immunol. *174*, 3891–3896.

Gause, W.C., Wynn, T.A., and Allen, J.E. (2013). Type 2 immunity and wound healing: evolutionary refinement of adaptive immunity by helminths. Nat. Rev. Immunol. *13*, 607–614.

Gauvreau, G.M., Boulet, L.P., Cockcroft, D.W., FitzGerald, J.M., Mayers, I., Carlsten, C., Laviolette, M., Killian, K.J., Davis, B.E., Larché, M., et al. (2014). OX40L blockade and allergen-induced airway responses in subjects with mild asthma. Clin. Exp. Allergy *44*, 29–37.

Gold, M.J., Antignano, F., Halim, T.Y., Hirota, J.A., Blanchet, M.R., Zaph, C., Takei, F., and McNagny, K.M. (2014). Group 2 innate lymphoid cells facilitate sensitization to local, but not systemic, TH2-inducing allergen exposures. J. Allergy Clin. Immunol. *133*, 1142–1148.

Gollwitzer, E.S., Saglani, S., Trompette, A., Yadava, K., Sherburn, R., McCoy, K.D., Nicod, L.P., Lloyd, C.M., and Marsland, B.J. (2014). Lung microbiota promotes tolerance to allergens in neonates via PD-L1. Nat. Med. *20*, 642–647.

Guo, L., Huang, Y., Chen, X., Hu-Li, J., Urban, J.F., Jr., and Paul, W.E. (2015). Innate immunological function of TH2 cells in vivo. Nat. Immunol. *16*, 1051–1059.

Halim, T.Y., Krauss, R.H., Sun, A.C., and Takei, F. (2012a). Lung natural helper cells are a critical source of Th2 cell-type cytokines in protease allergen-induced airway inflammation. Immunity *36*, 451–463.

Halim, T.Y., MacLaren, A., Romanish, M.T., Gold, M.J., McNagny, K.M., and Takei, F. (2012b). Retinoic-acid-receptor-related orphan nuclear receptor alpha is required for natural helper cell development and allergic inflammation. Immunity *37*, 463–474.

Halim, T.Y., Steer, C.A., Mathä, L., Gold, M.J., Martinez-Gonzalez, I., McNagny, K.M., McKenzie, A.N., and Takei, F. (2014). Group 2 innate lymphoid cells are critical for the initiation of adaptive T helper 2 cell-mediated allergic lung inflammation. Immunity *40*, 425–435.

Halim, T.Y., Hwang, Y.Y., Scanlon, S.T., Zaghouani, H., Garbi, N., Fallon, P.G., and McKenzie, A.N. (2016). Group 2 innate lymphoid cells license dendritic cells to potentiate memory TH2 cell responses. Nat. Immunol. *17*, 57–64.

Hammad, H., and Lambrecht, B.N. (2015). Barrier epithelial cells and the control of type 2 immunity. Immunity *43*, 29–40.

Heng, T.S., and Painter, M.W.; Immunological Genome Project Consortium (2008). The Immunological Genome Project: networks of gene expression in immune cells. Nat. Immunol. *9*, 1091–1094.

Hoshino, A., Tanaka, Y., Akiba, H., Asakura, Y., Mita, Y., Sakurai, T., Takaoka, A., Nakaike, S., Ishii, N., Sugamura, K., et al. (2003). Critical role for OX40 ligand in the development of pathogenic Th2 cells in a murine model of asthma. Eur. J. Immunol. *33*, 861–869.

Hoyler, T., Klose, C.S., Souabni, A., Turqueti-Neves, A., Pfeifer, D., Rawlins, E.L., Voehringer, D., Busslinger, M., and Diefenbach, A. (2012). The transcription factor GATA-3 controls cell fate and maintenance of type 2 innate lymphoid cells. Immunity *37*, 634–648.

Jember, A.G., Zuberi, R., Liu, F.T., and Croft, M. (2001). Development of allergic inflammation in a murine model of asthma is dependent on the costimulatory receptor OX40. J. Exp. Med. *193*, 387–392.

Jenkins, S.J., Perona-Wright, G., Worsley, A.G., Ishii, N., and MacDonald, A.S. (2007). Dendritic cell expression of OX40 ligand acts as a costimulatory, not polarizing, signal for optimal Th2 priming and memory induction in vivo. J. Immunol. *179*, 3515–3523.

Kim, M.Y., Anderson, G., White, A., Jenkinson, E., Arlt, W., Martensson, I.L., Erlandsson, L., and Lane, P.J. (2005). OX40 ligand and CD30 ligand are expressed on adult but not neonatal CD4+CD3- inducer cells: evidence that IL-7 signals regulate CD30 ligand but not OX40 ligand expression. J. Immunol. *174*, 6686–6691.

Lewkowich, I.P., Herman, N.S., Schleifer, K.W., Dance, M.P., Chen, B.L., Dienger, K.M., Sproles, A.A., Shah, J.S., Köhl, J., Belkaid, Y., and Wills-Karp, M. (2005). CD4+CD25+ T cells protect against experimentally induced asthma and alter pulmonary dendritic cell phenotype and function. J. Exp. Med. *202*, 1549–1561.

Linch, S.N., McNamara, M.J., and Redmond, W.L. (2015). OX40 agonists and combination immunotherapy: putting the pedal to the metal. Front. Oncol. *5*, 34.

Maazi, H., Patel, N., Sankaranarayanan, I., Suzuki, Y., Rigas, D., Soroosh, P., Freeman, G.J., Sharpe, A.H., and Akbari, O. (2015). ICOS:ICOS-ligand interaction is required for type 2 innate lymphoid cell function, homeostasis, and induction of airway hyperreactivity. Immunity *42*, 538–551.

Man, K., Kutyavin, V.I., and Chawla, A. (2017). Tissue immunometabolism: development, physiology, and pathobiology. Cell Metab. *25*, 11–26.

Mirchandani, A.S., Besnard, A.G., Yip, E., Scott, C., Bain, C.C., Cerovic, V., Salmond, R.J., and Liew, F.Y. (2014). Type 2 innate lymphoid cells drive CD4+ Th2 cell responses. J. Immunol. *192*, 2442–2448.

Mjösberg, J., Bernink, J., Golebski, K., Karrich, J.J., Peters, C.P., Blom, B., te Velde, A.A., Fokkens, W.J., van Drunen, C.M., and Spits, H. (2012). The transcription factor GATA3 is essential for the function of human type 2 innate lymphoid cells. Immunity *37*, 649–659.

Mohrs, M., Shinkai, K., Mohrs, K., and Locksley, R.M. (2001). Analysis of type 2 immunity in vivo with a bicistronic IL-4 reporter. Immunity *15*, 303–311.

Molofsky, A.B., Van Gool, F., Liang, H.E., Van Dyken, S.J., Nussbaum, J.C., Lee, J., Bluestone, J.A., and Locksley, R.M. (2015). Interleukin-33 and interferon-γ counter-regulate group 2 innate lymphoid cell activation during immune perturbation. Immunity *43*, 161–174.

Monticelli, L.A., Sonnenberg, G.F., Abt, M.C., Alenghat, T., Ziegler, C.G., Doering, T.A., Angelosanto, J.M., Laidlaw, B.J., Yang, C.Y., Sathaliyawala, T., et al. (2011). Innate lymphoid cells promote lung-tissue homeostasis after infection with influenza virus. Nat. Immunol. *12*, 1045–1054.

Moon, J.J., Chu, H.H., Pepper, M., McSorley, S.J., Jameson, S.C., Kedl, R.M., and Jenkins, M.K. (2007). Naive CD4(+) T cell frequency varies for different epitopes and predicts repertoire diversity and response magnitude. Immunity *27*, 203–213.

Morita, H., Arae, K., Unno, H., Miyauchi, K., Toyama, S., Nambu, A., Oboki, K., Ohno, T., Motomura, K., Matsuda, A., et al. (2015). An interleukin-33-mast cell-interleukin-2 axis suppresses papain-induced allergic inflammation by promoting regulatory T cell numbers. Immunity *43*, 175–186.

Nair, M.G., Du, Y., Perrigoue, J.G., Zaph, C., Taylor, J.J., Goldschmidt, M., Swain, G.P., Yancopoulos, G.D., Valenzuela, D.M., Murphy, A., et al. (2009). Alternatively activated macrophage-derived RELM-alpha is a negative regulator of type 2 inflammation in the lung. J. Exp. Med. *206*, 937–952.

Odegaard, J.I., and Chawla, A. (2015). Type 2 responses at the interface between immunity and fat metabolism. Curr. Opin. Immunol. *36*, 67–72.

Oliphant, C.J., Hwang, Y.Y., Walker, J.A., Salimi, M., Wong, S.H., Brewer, J.M., Englezakis, A., Barlow, J.L., Hams, E., Scanlon, S.T., et al. (2014). MHCII-mediated dialog between group 2 innate lymphoid cells and CD4(+) T cells potentiates type 2 immunity and promotes parasitic helminth expulsion. Immunity *41*, 283–295.

Panduro, M., Benoist, C., and Mathis, D. (2016). Tissue Tregs. Annu. Rev. Immunol. *34*, 609–633.

Pulendran, B., and Artis, D. (2012). New paradigms in type 2 immunity. Science *337*, 431–435.

Saluzzo, S., Gorki, A.D., Rana, B.M.J., Martins, R., Scanlon, S., Starkl, P., Lakovits, K., Hladik, A., Korosec, A., Sharif, O., et al. (2017). First-breath-induced type 2 pathways shape the lung immune environment. Cell Rep. *18*, 1893–1905.

Schiering, C., Krausgruber, T., Chomka, A., Fröhlich, A., Adelmann, K., Wohlfert, E.A., Pott, J., Griseri, T., Bollrath, J., Hegazy, A.N., et al. (2014). The alarmin IL-33 promotes regulatory T-cell function in the intestine. Nature *513*, 564–568.

Schlenner, S.M., Madan, V., Busch, K., Tietz, A., Läufle, C., Costa, C., Blum, C., Fehling, H.J., and Rodewald, H.R. (2010). Fate mapping reveals separate origins of T cells and myeloid lineages in the thymus. Immunity *32*, 426–436.

Schwartz, C., Khan, A.R., Floudas, A., Saunders, S.P., Hams, E., Rodewald, H.R., McKenzie, A.N.J., and Fallon, P.G. (2017). ILC2s regulate adaptive Th2 cell functions via PD-L1 checkpoint control. J. Exp. Med. *214*, 2507–2521.

Siede, J., Fröhlich, A., Datsi, A., Hegazy, A.N., Varga, D.V., Holecska, V., Saito, H., Nakae, S., and Löhning, M. (2016). IL-33 receptor-expressing regulatory T cells are highly activated, Th2 biased and suppress CD4 T cell proliferation through IL-10 and TGFβ release. PLoS ONE *11*, e0161507.

Steer, C.A., Martinez-Gonzalez, I., Ghaedi, M., Allinger, P., Mathä, L., and Takei, F. (2017). Group 2 innate lymphoid cell activation in the neonatal lung drives type 2 immunity and allergen sensitization. J. Allergy Clin. Immunol. *140*, 593–595.e3.

Suto, A., Nakajima, H., Kagami, S.I., Suzuki, K., Saito, Y., and Iwamoto, I. (2001). Role of CD4(+) CD25(+) regulatory T cells in T helper 2 cell-mediated allergic inflammation in the airways. Am. J. Respir. Crit. Care Med. *164*, 680–687.

Tahiliani, V., Hutchinson, T.E., Abboud, G., Croft, M., and Salek-Ardakani, S. (2017). OX40 cooperates with ICOS to amplify follicular Th cell development and germinal center reactions during infection. J. Immunol. *198*, 218–228.

Takeda, I., Ine, S., Killeen, N., Ndhlovu, L.C., Murata, K., Satomi, S., Sugamura, K., and Ishii, N. (2004). Distinct roles for the OX40-OX40 ligand interaction in regulatory and nonregulatory T cells. J. Immunol. *172*, 3580–3589.

Taylor, M.D., van der Werf, N., and Maizels, R.M. (2012). T cells in helminth infection: the regulators and the regulated. Trends Immunol. *33*, 181–189.

Thornton, A.M., Korty, P.E., Tran, D.Q., Wohlfert, E.A., Murray, P.E., Belkaid, Y., and Shevach, E.M. (2010). Expression of Helios, an Ikaros transcription factor family member, differentiates thymic-derived from peripherally induced Foxp3+ T regulatory cells. J. Immunol. *184*, 3433–3441.

Van Dyken, S.J., Nussbaum, J.C., Lee, J., Molofsky, A.B., Liang, H.E., Pollack, J.L., Gate, R.E., Haliburton, G.E., Ye, C.J., Marson, A., et al. (2016). A tissue checkpoint regulates type 2 immunity. Nat. Immunol. *17*, 1381–1387.

Vasanthakumar, A., Moro, K., Xin, A., Liao, Y., Gloury, R., Kawamoto, S., Fagarasan, S., Mielke, L.A., Afshar-Sterle, S., Masters, S.L., et al. (2015). The transcriptional regulators IRF4, BATF and IL-33 orchestrate development and maintenance of adipose tissue-resident regulatory T cells. Nat. Immunol. *16*, 276–285.

Vély, F., Barlogis, V., Vallentin, B., Neven, B., Piperoglou, C., Ebbo, M., Perchet, T., Petit, M., Yessaad, N., Touzot, F., et al. (2016). Evidence of innate lymphoid cell redundancy in humans. Nat. Immunol. *17*, 1291–1299.

von Burg, N., Chappaz, S., Baerenwaldt, A., Horvath, E., Bose Dasgupta, S., Ashok, D., Pieters, J., Tacchini-Cottier, F., Rolink, A., Acha-Orbea, H., and Finke, D. (2014). Activated group 3 innate lymphoid cells promote T-cell-mediated immune responses. Proc. Natl. Acad. Sci. USA *111*, 12835–12840.

Webb, G.J., Hirschfield, G.M., and Lane, P.J. (2016). OX40, OX40L and autoimmunity: a comprehensive review. Clin. Rev. Allergy Immunol. *50*, 312–332.

Wohlfert, E.A., Grainger, J.R., Bouladoux, N., Konkel, J.E., Oldenhove, G., Ribeiro, C.H., Hall, J.A., Yagi, R., Naik, S., Bhairavabhotla, R., et al. (2011). GATA3 controls $Foxp3^+$ regulatory T cell fate during inflammation in mice. J. Clin. Invest. *121*, 4503–4515.

Wong, S.H., Walker, J.A., Jolin, H.E., Drynan, L.F., Hams, E., Camelo, A., Barlow, J.L., Neill, D.R., Panova, V., Koch, U., et al. (2012). Transcription factor RORα is critical for nuocyte development. Nat. Immunol. *13*, 229–236.

Yadav, M., Louvet, C., Davini, D., Gardner, J.M., Martinez-Llordella, M., Bailey-Bucktrout, S., Anthony, B.A., Sverdrup, F.M., Head, R., Kuster, D.J., et al. (2012). Neuropilin-1 distinguishes natural and inducible regulatory T cells among regulatory T cell subsets in vivo. J. Exp. Med. *209*, 1713–1722, S1711–1719.

Zheng, W., and Flavell, R.A. (1997). The transcription factor GATA-3 is necessary and sufficient for Th2 cytokine gene expression in CD4 T cells. Cell *89*, 587–596.

STAR★METHODS

KEY RESOURCES TABLE

REAGENT or RESOURCE	SOURCE	IDENTIFIER
Antibodies		
Anti-mouse CD3ε	Biolegend	100320; RRID: AB_312685
Anti-mouse CD4	Thermo Fisher	56-0041-82; RRID: AB_493999
Anti-mouse CD5	Thermo Fisher	48-0051-82; RRID: AB_1603250
Anti-mouse CD8α	Biolegend	100750; RRID: AB_2562610
Anti-mouse CD11b	Thermo Fisher	48-0112-82; RRID: AB_1582236
Anti-mouse CD11c	Thermo Fisher	48-0114-82; RRID: AB_1548654
Anti-mouse CD19	Thermo Fisher	48-0193-82; RRID: AB_2043815
Anti-mouse CD45	Biolegend	103137; RRID: AB_2561392
Anti-mouse CD127	BD Bioscience	562419; RRID: AB_11153131
Anti-mouse Gr-1	Thermo Fisher	48-5931-82; RRID: AB_1548788
Anti-mouse FcεR1α	Thermo Fisher	48-5898-82; RRID: AB_2574086
Anti-mouse Ter119	Thermo Fisher	48-5921-82; RRID: AB_1518808
Anti-mouse Foxp3	Thermo Fisher	53-5773-82; RRID: AB_763537
Anti-mouse RORγt	Thermo Fisher	46-6981-82; RRID: AB_10717956
Anti-mouse GATA3	Thermo Fisher	50-9966-42; RRID: AB_10596663
Anti-mouse NK1.1	BD Bioscience	56144
Anti-mouse CTLA4	Biolegend	106305; RRID: AB_313254
Anti-mouse MHCII	Thermo Fisher	48-5321-82; RRID: AB_1272204
Anti-mouse Ly6B (7/4)	Abcam	Ab53453; RRID: AB_881408
Anti-mouse OX40	Biolegend	119409; RRID: AB_2272150
Anti-mouse OX40L	Thermo Fisher	12-5905-82; RRID: AB_466036
Anti-mouse TCRβ	Thermo Fisher	48-5961-82; RRID: AB_11039532
Anti-mouse Neuropillin1	Biolegend	145204; RRID: AB_2561928
Anti-mouse Helios	Biolegend	137204; RRID: AB_10549181
Anti-mouse Ki67	Thermo Fisher	25-5698-82; RRID: AB_11220070
Anti-mouse Siglec-F	BD Bioscience	552126; RRID: AB_39431
Anti-mouse F4/80	Thermo Fisher	48-4801-82; RRID: AB_1548747
Anti-mouse IL-13	Thermo Fisher	25-7133-82; RRID: AB_2573530
Anti-mouse B220	Thermo Fisher	47-0452-82; RRID: AB_1518810
Anti-mouse RELMα	Peprotech	500-p214; RRID: AB_1268707
Anti-mouse CD3ε (eBio500A2)	Thermo Fisher	14-0033-82; RRID: AB_837128
Anti-mouse Foxp3 (TWAJ)	Thermo Fisher	53-9966-42; RRID: AB_2574493
Anti-mouse CD127 (A7R34)	Thermo Fisher	17-1271-82; RRID: AB_469435
Anti-mouse IFNγ	Biolegend	505837; RRID: AB_11219004
Anti-human CD2	Thermo Fisher	17-0029-42; RRID: AB_10805740
Anti-mouse ST2	Thermo Fisher	46-9333-82; RRID: AB_2573881
Donkey anti-rat IgG FITC	Jackson ImmunoResearch	712-095-150; RRID: AB_2340651
Rabbit anti-FITC AF488	Life Technologies	A-11090; RRID: AB_221562
Donkey anti-rabbit IgG AF488	Life Technologies	A-21206; RRID: AB_141708
Anti-mouse CD40	Thermo Fisher	16-0401-82; RRID: AB_468941
IgG isotype control	Bio X Cell	BE0085; RRID: AB_1107771
Anti-human CD8α	Biolegend	301014; RRID: AB_314132
Anti-human CD11b	Biolegend	301309; RRID: AB_314161

(*Continued on next page*)

Continued		
REAGENT or RESOURCE	SOURCE	IDENTIFIER
Anti-human CD11c	Biolegend	337207; RRID: AB_1279068
Anti-human CD123	Biolegend	306011; RRID: AB_439778
Anti-human CD20	Biolegend	302309; RRID: AB_314257
Anti-human CD56	Biolegend	318309; RRID: AB_604098
Anti-human CD71	Biolegend	334107; RRID: AB_10916388
Anti-human FcεR1α	Biolegend	334612; RRID: AB_10578086
Anti-human CD45	Biolegend	304023; RRID: AB_493760
Anti-human CD127	Biolegend	351335; RRID: AB_2563636
Anti-human CRTH2	Biolegend	350105; RRID: AB_10900255
Anti-human OX40L	Biolegend	326307; RRID: AB_2207272
Biological Samples		
Fresh human PBMC obtained from healthy anonymous adult donors. No additional information is available, and was not required for the experimental design.	N/A	N/A
Chemicals, Peptides, and Recombinant Proteins		
Recombinant mouse IL-25	Janssen	N/A
Recombinant mouse IL-33	Biolegend	580508
Recombinant mouse TSLP	Thermo Fisher	34-8498-82
Recombinant mouse TSLP	R&D Systems	555-TS-010
Recombinant mouse IL-2	Biolegend	575406
Recombinant mouse IL-7	Biolegend	577806
Recombinant human IL-33	Biolegend	581802
Recombinant human IL-2	Biolegend	589102
Recombinant human IL-7	Biolegend	581902
2W1S peptide (EAWGALANWAVDSA)	Designer BioScience	N/A
I-A(b) 2W1S tetramer	NIH Tetramer Core	N/A
Foxp3 intracellular staining kit	Thermo Fisher	88-8008-74
Protein transport inhibitor cocktail	Thermo Fisher	00-4980-03
Lipopolysaccharide (LPS)	Sigma	L3129-10MG
Alternaria alternata extract	Greer Laboratories	Alternaria alternata, Alternaria tenuis (My1)
Papain	Sigma	76216
Diphtheria toxin	Merck	322326-1MG
DRAQ7	Biolegend	424001
eFluor 780 viability dye	Thermo Fisher	65-0865-14
eFluor 455 (UV) Viability dye	Thermo Fisher	65-0868-14
Streptavidin AF555	Life Technologies	S32355
Lymphoprep	STEMCELL Tech	07801
TruStain FcX	Biolegend	422301
M-280 Dynabeads	Thermo Fisher	11205D
O.C.T. compound	Tissue-Tek	23-730-571
Collagenase I	Life Technologies	17100017
DNase I	Quiagen	79254
Percoll	Sigma	GE17-0891-09
UltraComp beads	Invitrogen	01-2222-42
Precision Count Beads	Biolegend	424902
BCA Protein Assay kit	Thermo	23227
Ovation Ultralow System V2 1-16	Nugen	0344NB
Ovation RNA-Seq System V2	Nugen	7102

(*Continued on next page*)

Continued

REAGENT or RESOURCE	SOURCE	IDENTIFIER
RNA 6000 Pico Chip	Agilent	5067-1513
High sensitivity DNA Chip	Agilent	5067-4626
Critical Commercial Assays		
Magpix array (IL-4, IL-5)	Millipore	MCYTOMAG-70K
$CD4^+$ T cell enrichment kit	Miltenyi	130-104-453
Murine IgE ELISA	Thermo Fisher	88-50460-88
Deposited Data		
RNA-seq data	This paper	GEO: GSE112937
Microarray data (lung ILC2)	Halim et al., 2012a	GEO: GSE36057
Microarray data (other)	Heng et al., 2008	GEO: GSE15907 and GSE37448
Experimental Models: Organisms/Strains		
Mouse: C57BL/6 (B6)	Charles River	027
Mouse: B6.*Rag2*$^{-/-}$	JAX	008449; RRID: IMSR_JAX:008449
Mouse: B6.*Il1rl1*$^{-/-}$	Dr. ANJ McKenzie	N/A
Mouse: B6.iCOS-T (B6.*Icos*$^{fl\text{-}DTR\text{-}fl/+}$*Cd4*$^{Cre/+}$)	Dr. ANJ McKenzie	(Oliphant et al., 2014)
Mouse: B6.*Tnfrsf4*$^{-/-}$	Prof. P Lane	N/A
Mouse: B6.*Il7r*$^{Cre/+}$	Prof. HR Rodewald	(Schlenner et al., 2010)
Mouse: B6.*Il7r*$^{Cre/+}$*Rora*$^{fl/fl}$	Dr. ANJ McKenzie	N/A
Mouse: B6.*Rorc*$^{Cre/+}$*Tnfsf4*$^{fl/fl}$	Dr. D Withers	N/A
Mouse: B6.*Rorc*$^{Cre/+}$	Dr. D Withers	N/A
Mouse: B6.*Tnfsf4*$^{fl/fl}$	Prof. M Botto and Prof. TJ Vyse	(Cortini et al., 2017)
Mouse: B6.*Il7r*$^{Cre/+}$*Tnfsf4*$^{fl/fl}$	Dr. ANJ McKenzie	N/A
Mouse: B6.*Cd4*^{Cre}*Tnfsf4*$^{fl/fl}$	Dr. ANJ McKenzie	N/A
Mouse: B6.*Cd4*^{Cre}	Dr. ANJ McKenzie	N/A
Mouse: B6.*Cd4*^{Cre}*Rora*$^{fl/fl}$	Dr. SA Teichmann	N/A
Mouse: B6.*Foxp3*$^{DTR\text{-}egfp/+}$*Gata3*$^{hCD2/+}$	Dr. ANJ McKenzie	N/A
Mouse: B6.Cg-$^{Tg(Itgax\text{-}EGFP\text{-}CRE\text{-}DTR\text{-}LUC)2Gjh/Crl}$ (*Itgax*$^{Cre/+}$)	Prof. N Garbi	N/A
Mouse: B6.*Itgax*$^{Cre/+}$*Tnfsf4*$^{fl/fl}$	Dr. ANJ McKenzie	N/A
Mouse: B6. *Ighm*$^{tm1Cgn/J}$ (μMT)	Dr. ANJ McKenzie	N/A
Mouse: B6.*Il33*$^{cit/cit}$ (*Il33*$^{-/-}$)	Dr. ANJ McKenzie	N/A
Software and Algorithms		
Flowjo V10	FlowJo, LLC	https://www.flowjo.com/
FlexArray 1.5	Genome Quebec	http://www.genomequebec.com/en/home.html
Partek Flow	Partek	http://www.partek.com
Partek Genomics Suite	Partek	http://www.partek.com
GraphPad Prism 7	GraphPad Software, Inc	http://www.graphpad.com/scientific-software/prism/

CONTACT FOR REAGENT AND RESOURCE SHARING

Further information and requests for resources and reagents should be directed to and will be fulfilled by the Lead Contact, Tim Halim (tim.halim@cruk.cam.ac.uk).

EXPERIMENTAL MODEL AND SUBJECT DETAILS

In vivo animal studies

C57BL/6 (B6), B6.*Il33*$^{cit/cit}$ (*Il33*$^{-/-}$), B6.*Il1rl1*$^{-/-}$, B6.*Tnfrsf4*$^{-/-}$, B6.*Il7r*$^{Cre/+}$ (Schlenner et al., 2010), B6.*Il7r*$^{Cre/+}$*Rora*$^{fl/fl}$, B6.*Rorc*$^{Cre/+}$*Tnfsf4*$^{fl/fl}$, B6.*Tnfsf4*$^{fl/fl}$ mice (Cortini et al., 2017), B6.*Il7r*$^{Cre/+}$*Tnfsf4*$^{fl/fl}$, B6.*Foxp3*$^{DTR\text{-}egfp/+}$, B6.*Cd4*^{Cre}*Tnfsf4*$^{fl/fl}$,

B6.$Cd4^{Cre}Rora^{fl/fl}$, B6.$Foxp3^{DTR-egfp/+}Gata3^{hCD2/+}$ (B6.$Gata3^{hCD2/+}$ mice, manuscript in preparation, J.A.W. and A.N.J.M), B6.Cg-$^{Tg(Itgax-EGFP-CRE-DTR-LUC)2Gjh/Crl}$, B6.$Itgax^{Cre/+}Tnfsf4^{fl/fl}$, B6.iCOS-T (B6.$Icos^{fl-DTR-fl/+}Cd4^{Cre/+}$) (Oliphant et al., 2014), B6.$Ighm^{tm1Cgn/J}$, B6.$Rag2^{-/-}$ and B6.$Rorc^{Cre/+}$ mice were maintained in the Medical Research Council (MRC) ARES animal facility, under specific-pathogen-free conditions. Animals were sex and age matched whenever possible, and most animals were used at 6-10 weeks of age (we used OX40$^{-/-}$ mice that were over 12 weeks old, which have normal Treg cell number during homeostasis (Takeda et al., 2004). All animal use was in accordance with the guidelines of the UK Home Office.

Human studies
Peripheral blood samples were obtained from healthy anonymous adult donors after written informed consent was given. Whole blood was subjected to density centrifugation on Lymphoprep (STEMCELL Technologies) and peripheral blood mononucleated cells (PBMC) were collected from the interphase. All human work was approved by the appropriate UK research ethics committees (15/EE/0408).

Primary cell culture
Human and mouse primary cells were cultured at 37°C in a humidified, 5% CO_2 incubator.

METHOD DETAILS

Primary leukocyte preparation
Cell suspensions were prepared from lung by mechanical dissociation, followed by digest in 3 mL of RPMI-1640 containing collagenase I (500 U/ml) and DNase I (0.2 mg/ml) for 45 minutes at 37°C on a shaker (220 rpm), followed by filtration through a 70 μm strainer and 25% Percoll gradient enrichment of leukocytes and red blood cell (RBC) lysis. LN was strained through a 70 μm strainer with PBS, followed by RBC lysis. BAL cells and fluid were obtained in PBS as described (Halim et al., 2014). Cell suspensions were prepared from perigonadal adipose tissue by mechanical dissociation, followed by digest in 1 mL of RPMI-1640 containing collagenase I (500 U/ml) and DNase I (0.2 mg/ml) for 45 minutes at 37°C on a shaker (1100 rpm), followed by filtration through a 70 μm strainer and RBC lysis. Cell suspensions were prepared from large intestine by initial cleaning with PBS (10 mM HEPES) and mechanical dissociation, incubation in EDTA/DTT to remove intraepithelial cells, followed by digest in 8 mL of RPMI-1640 (10 mM HEPES) containing Liberase (0.0625 mg/ml, Roche) and DNase I (0.2 mg/ml) for 30 minutes at 37°C on a shaker (200 rpm), followed by filtration through a 70 μm strainer and Percoll gradient enrichment of leukocytes and red blood cell (RBC) lysis. Single-cells were re-stimulated and stained for surface and intracellular markers as described below.

Flow cytometry
Single cells were incubated with anti-mouse CD16/32 (Thermo Fisher) to block Fc receptors and stained as indicated. Lineage cocktail contained (±CD3, ± NK1.1, TCRβ, CD5, CD19, CD11b, CD11c, FcεR1α, F4/80, Ly-6C/G, and Ter119). For intracellular staining we used the Foxp3/Transcription Factor Kit (Thermo Fisher). For intracellular cytokine detection, single cells were stimulated with PMA (60 ng/ml) and ionomycin (500 ng/ml) and 1X protein transport inhibitor (Thermo Fisher) in culture media (RPMI-1640, 10% FCS) at 37°C for 3 hours before staining. Flow cytometry analysis was performed on a BD Fortessa instrument. Cells were quantified using CountBright beads or manual cell counting by hemocytometer. Flow cytometry data was analyzed using FlowJo X (Tree Star).

In vivo stimulation
Mice were anesthetized by isofluorane inhalation, followed by the intranasal administration of rmIL-25 (0.2 μg), rmIL-33 (0.2 μg), rmTSLP (0.2 μg), LPS, *Alternaria alternata* extract (10 μg, Greer Laboratories), or papain (10 μg) in 40 μl of PBS. Diphtheria toxin (300–500 ng), anti-CD40 mAb or IgG control (100 μg), or rmIL-33 (0.5 μg) was administered by intraperitoneal injection in 200 μl PBS. For *Nippostrongylus brasiliensis* infections 500 live L3 larvae were injected subcutaneously in 100 μl of water.

Adoptive CD4$^+$ T cell transfer
CD4$^+$ T cells were enriched from naive B6 and B6.$Il1rl1^{-/-}$ mice using a magnetic bead negative selection strategy (Miltenyi Biotech). CD4$^+$ T cell enriched cells were quantified and equal numbers of each genotype was adoptively transferred to CD45.1 congenic B6.$Rag2^{-/-}$ recipient mice on day −5. Adoptively transferred mice where subsequently used as indicated.

Bone marrow transplantation
Recipient mice were lethally irradiated (2 doses of 4.5 Gy) followed by intravenous transplantation of 10^7 whole bone marrow cells from 4-6 week old mice. Mice were given Baytril in drinking water for 4 weeks, and used for analysis at 24-32 weeks post transplant.

IF microscopy
Lungs were infused with 50% O.C.T. (Tissue-Tek) in PBS immediately after mice were killed. Lungs were subsequently frozen in 100% O.C.T. compound, followed by sectioning. 6 μm-thick sections of frozen lung tissue were cut, fixed in cold acetone at 4 °C for 20 min and then stored at −20 °C before staining. Antibodies raised against the following mouse antigens were used: CD3 (biotinylated, clone eBio500A2, Thermo Fisher), FoxP3 (APC conjugated, clone FJK-16 s, Thermo Fisher), GATA-3 (unconjugated, clone

TWAJ, Thermo Fisher), IL-7Rα (efluor660 conjugated, clone A7R34, Thermo Fisher). Detection of GATA-3 expression required amplification of the signal: the primary antibodies were detected with donkey anti-rat-IgG-FITC (Jackson ImmunoResearch), then rabbit anti-FITC-AF488 (Life Technologies) and then with donkey anti-rabbit-IgG-AF488 (Life Technologies). Biotinylated anti-CD3 antibodies were detected with SA-AF555 (Life Technologies). Sections were counterstained with DAPI (Invitrogen) and mounted using ProLong Gold (Invitrogen). Slides were analyzed on a Zeiss 780 Zen microscope (Zeiss).

Histology

All mouse lung lobes were weighed and a single lobe was used for histology. Lung lobes were fixed in 10% formaldehyde in PBS for 24 hours at 4°C, followed by transfer to 70% Ethanol in PBS for 24 hours at 4°C. Finally, lung lobes were transferred to PBS and kept at 4°C until paraffin embedding. Sections were cut and stained with Mason's trichrome reagent. The CRUK Cambridge Institute Histology Core performed tissue embedding, sectioning and staining.

Human ILC2 culture

PBMC were blocked with TruStain FcX and stained with biotinylated αCD3, αCD14 and αCD19 for the depletion of T cells, monocytes and B cells by MACS using M-280 streptavidin Dynabeads (Thermo Fisher), according to the manufacturer's instructions. Following further antibody staining, ILC2 were sorted from depleted PBMC based on surface marker expression as lineage negative (streptavidin-APC, CD8a, CD11b, CD11c, FcεRIα, CD123, CD20, CD56, CD71), DRAQ7$^-$, CD45$^+$, CD127$^+$ and CRTH2$^+$ on a BD Influx. Sorted ILC2 were cultured in RPMI (GIBCO) supplemented with 10% FBS, 100 μM 2-mercaptoethanol, 100 μg/mL penicillin, 100 IU/mL streptomycin, 20 mM HEPES and 10 ng/mL recombinant human IL-2, IL-7 and IL-33 (as indicated). OX40L surface protein expression was assessed by flow cytometry after 72 hours of culture. Briefly, following Fc-receptor blocking, ILC2 were stained with lineage antibodies (CD3, CD11b, CD19), OX40L and efluor780 fixable viability dye followed by acquisition on a BD LSR Fortessa flow cytometer. Data was analyzed with FlowJo 10.2.

Gene expression analysis

We obtained microarray datasets for the listed cell-types (T.8Nve.Sp1/2, T.4Nve.SP5/6, T.4Mem4h62l.Sp1.2, T.4FP3+25+.Sp2/3, B.Fo.Sp, MC.PC4/5, MF.11c-11b+.Lu, Mo.6C-II-Lu.v2, DC.103-11b+24+.Lu, GN.Bl, Eo.BL.v2, NKT.4+.SP1/2, NK.Sp7/8, ILC1.CD127+.SP1/2, BA.SP1/3, LPL.NCR+ILC3.1/2,) from data assembled by the ImmGen consortium (Heng et al., 2008), which was compared to our own lung ILC2 microarray data (Halim et al., 2012a). Data analysis was performed with FlexArray 1.5 (Genome Quebec).

IgE measurement

Lungs were frozen in liquid nitrogen, then homogenized in PBS protein inhibitor (cOmplete EDTA free protease inhibitor tablets, Roche), centrifuged to remove cell debris and supernatants were harvested. Total lung protein was measured using a BCA assay (Thermo Fisher) and Lung and serum IgE concentrations were measured by ELISA assay (Thermo Fisher).

RNA-seq

Foxp3^{egfp+}GATA3^{hDC2+} mice were treated with nothing or rmIL-33 (0.2 μg) in PBS (i/n, day 0 and 1) followed by collection of lung tissue on day 5. Single cells were incubated with anti-mouse CD16/32 to block Fc receptors and stained with DAPI, anti-CD45, CD3, CD4, B220, Lineage (NK1.1, Gr-1, CD11b, CD11b, Ter119, CD19, CD5, F4/80, FcεR1α), and hCD2 mAb, followed by sorting of: DAPI (Live)$^-$ CD45$^+$ B220$^-$ into CD3$^+$ CD4$^+$ eGFP(Foxp3)$^{+/-}$ hCD2(Gata3)$^{+/-}$ T cells, and DAPI(Live)$^-$ CD45$^+$ B220$^-$ Lineage$^-$ CD3$^-$ CD4$^-$ hCD2(Gata3)$^+$ ILC2. CLP were sorted from the bone marrow as CD45$^+$ Lineage$^-$ Sca-1^{int} CD127$^+$ Flt3$^+$ cells. Cells were flow sorted into PBS 2% FCS and transferred to Trizol LS (Life Technologies). chloroform extractions were performed and 1.5X volume of 100% ethanol was added to the aqueous phase. RNA was extracted using RNeasy kit (QIAGEN), gDNA was digested using Turbo DNase (Ambion), concentrated using RNeasy Micro Kit (QIAGEN) and assessed using the Bioanalyser (Agilent). RNA was processed for RNA sequencing using Ovation RNA-seq System V2 (Nugen), fragmented using the Covaris M220 and barcoded using Ovation Ultralow Library Systems (Nugen). Samples were sequenced using the Illumina Hiseq4000 (CRUK Cambridge Institute) and aligned using Tophat2 with Partek Flow. RPKM and differential gene transcript expression were generated using Genomics Suite® software, version 6.16 Copyright ©; 2016 Partek® Inc., St. Louis, MO, USA.

QUANTIFICATION AND STATISTICAL ANALYSIS

Statistics

Data were analyzed using GraphPad Prism 6 (GraphPad Software). A Kruskal-Wallis one-way ANOVA with Bonferroni post-analysis, or two-tailed Student's t test was used to determine the statistical significance between groups with $p \leq 0.05$ being considered significant (*), $p \leq 0.01$ = **, $p \leq 0.001$ = ***, $p \leq 0.0001$ = ****.

Additional details for main figures

Figure 1: (**A-C**, 3 repeat experiments, mean % gated population in **A**), (**D**, single experiment), (**E**, n = 4,4,4,4 left to right, ANOVA, 3 repeat experiments), (**F**, n = 5,5,5 left to right, ANOVA, 2 repeat experiments), (**G**, n = 4,4,4,4 left to right, ANOVA, 3 repeat experiments)

Figure 2: (**A**, 2 independent datasets per group), (**B-C**, n = 5,5,5,5 left to right in **C**, ANOVA, 2 repeat experiments), (**D-F**, n = 10,9,10 left to right in **D**, n = 10,10,10 in **E** and **F**, ANOVA, 2 pooled repeat experiments), (**G-I**, n = 5,5,5 left to right, ANOVA, 2 repeat experiments)

Figure 3: (**A**, 2 independent datasets per group), (**B**, n = 3, mean percent gated shown ± S.D., 3 repeat experiments), (**C**, n = 3,3,3,3,3,3 left to right, ANOVA, 2 repeat experiments), (**D**, n = 4,4,4 left to right, ANOVA, 2 repeat experiments), (**E**, n = 4, percent gated shown ± SD two-tailed Student's t test, 2 repeat experiments)

Figure 4: (**A**, n = 4,4,4 left to right, percent gated shown ± S.D., 3 repeat experiments), (**B-D**, n = 4,8,8,8 left to right for **B** and **C** n = 4,4,4,4 left to right for **D**, ANOVA, 2 repeat experiments), (**E-G**, n = 5,5, percent gated shown ± SD for **E**, n = 8,8,8,8 for **F** and **G** (n = 8,8,8,7 for Adipose), ANOVA, 2 pooled repeat experiments)

Figure 5: (**A**, n = 10,10,10 left to right, ANOVA, 2 pooled repeat experiments), (**B**, n = 10,10,10 left to right, ANOVA, 2 pooled repeat experiments), (**C**, n = 5,5,5 left to right, ANOVA, 2 repeat experiments), (**D** and **E**, n = 10,10,10 left to right, ANOVA, 2 pooled repeat experiments)

Figure 6: (**A-D**, n = 5,5,5,3,5 left to right, ANOVA, 2 repeat experiments), (**E**, n = 11,9,10 left to right, ANOVA, 3 repeat experiments (2 pooled experiments shown)), (**F-G**, n = 5,4,5 left to right, ANOVA, 2 repeat experiments, representative gate shown in **F**), (**H-I**, n = 5,5,5 left to right, ANOVA, 2 repeat experiments)

Figure 7: (**A-C**, n = 10,10,10,10 left to right, ANOVA, 3 repeat experiments (2 pooled experiments shown)), (**D**, 2 repeat experiments), (**E-I**, n = 5,5,5,5 left to right, ANOVA, 2 repeat experiments), (**J-L**, n = 10,10,10,10 left to right, ANOVA, 3 repeat experiments (2 pooled experiments shown)), (**M**, n = 5,5,5,5 left to right, ANOVA, 2 repeat experiments), (**N**, n = 10,13 left to right, two-tailed Student's t test, 2 pooled experiments)

DATA AND SOFTWARE AVAILABILITY

Microarray datasets for ILC2 are available under accession number GSE36057; all other microarray datasets are available under GSE15907 and GSE37448. RNA-seq datasets are available in GEO (GSE112937).

Immunity, Volume *48*

Supplemental Information

Tissue-Restricted Adaptive Type 2 Immunity Is Orchestrated by Expression of the Costimulatory Molecule OX40L on Group 2 Innate Lymphoid Cells

Timotheus Y.F. Halim, Batika M.J. Rana, Jennifer A. Walker, Bernhard Kerscher, Martin D. Knolle, Helen E. Jolin, Eva M. Serrao, Liora Haim-Vilmovsky, Sarah A. Teichmann, Hans-Reimer Rodewald, Marina Botto, Timothy J. Vyse, Padraic G. Fallon, Zhi Li, David R. Withers, and Andrew N.J. McKenzie

Supplemental Figures and Figure Legends

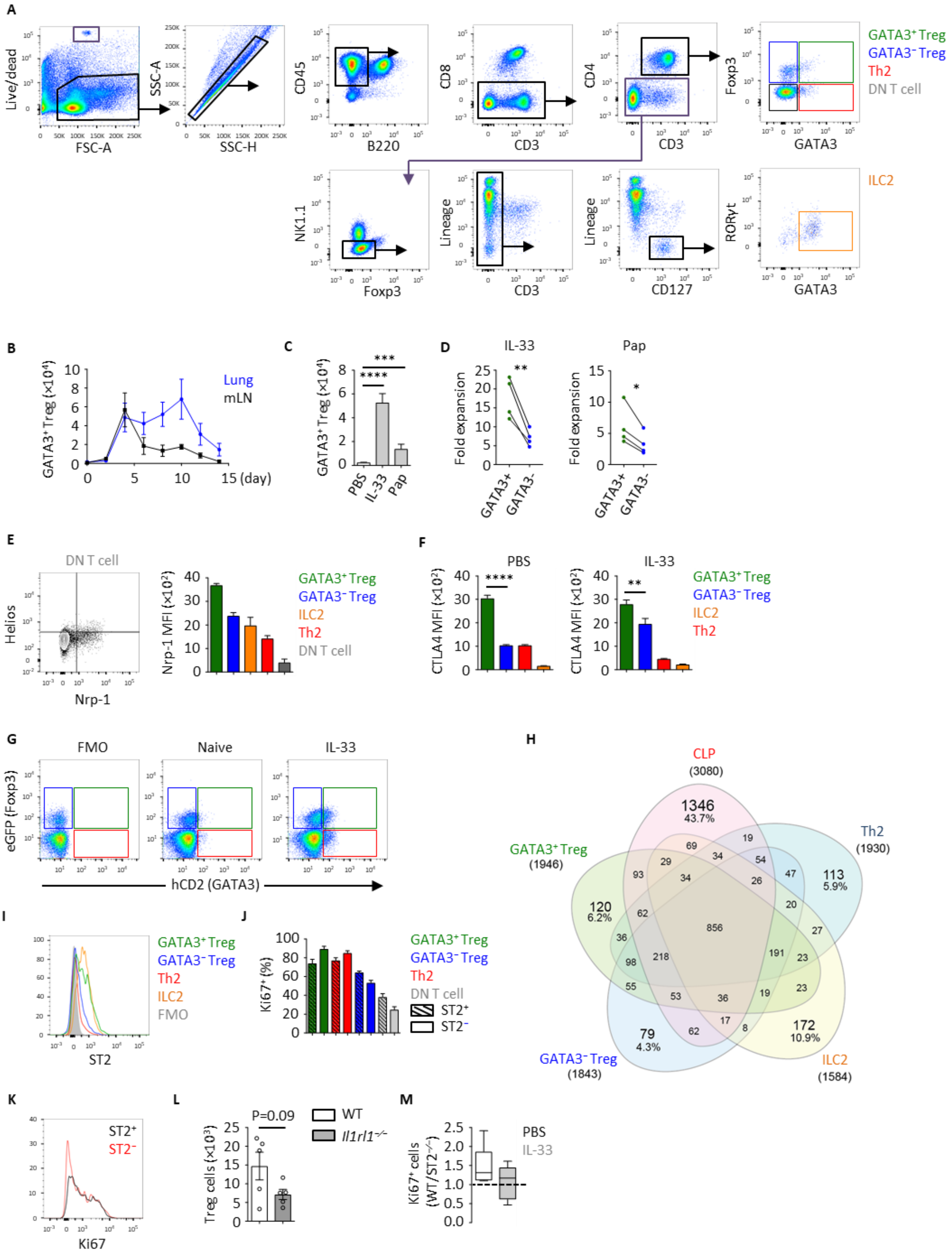

Figure S1 (Related to Figure 1)

A) A representative flow cytometry gating strategy for identifying and analysing adaptive type-2 lymphocytes. Live cells were identified by fixable viability dye exclusion. CD45$^+$B220$^-$CD8$^-$CD3$^+$CD4$^+$ (CD4) T cells were gated based on intracellular expression of Foxp3 and GATA3. CD45$^+$B220$^-$CD8$^-$CD3$^-$CD4$^-$NK1.1$^-$Lineage$^-$CD127$^+$ innate lymphoid

cells (ILC) were gated based on expression of RORγt and GATA3. These cells were subsequently analysed for other surface or intracellular markers;

B) WT mice were exposed to papain (i.n., day 0 and 1) followed by quantification of GATA3^{+} Foxp3^{+} Treg cells in the lung and mediastinal lymph node (mLN).

C) WT mice were exposed to papain (Pap) or IL-33 (i.n., day 0 and 1) followed by quantification of GATA3^{+}Foxp3^{+} Treg cells in the lung on day 5.

D) Fold expansion of GATA3^{+}Foxp3^{+} and GATA3^{+}Foxp3^{+} Treg cells in the lungs on day 5 of IL-33 and papain (Pap) treated mice (i.n., day 0 and 1). Fold expansion over PBS treated control groups was measured for each experiment (4-6 mice per group, average values of 4 individual experiments are shown, paired groups for each individual experiment are shown).

E) The indicated populations (see **Fig S1A**) were analysed for expression of Helios and Nrp-1. Mean fluorescence intensity of Nrp-1 was measured. Flow cytometry data for DN T cells (Foxp3^{-}GATA3^{-}, see a) is shown.

F) The indicated populations (see **Fig S1A**) were analysed for intracellular staining for CTLA4 on day 5 in PBS or IL-33 treated mice (i.n., day 0 and 1).

G) Lung ILC2, GATA3^{+} and GATA3^{-} Treg and Th2 cells were FACS purified on day 5 from *Foxp3*$^{EGFP\text{-}DTR/+}$*Gata3*$^{hCD2/+}$ mice treated with PBS or IL-33 (i.n., day 0 and 1). Shown is the gating profile of eGFP (*Foxp3*) and anti-hCD2 (*Gata3*) staining, and the FMO control.

H) Gene expression of naïve Foxp3^{egfp+}GATA3^{hDC2+} and Foxp3^{egfp+}GATA3^{hDC2-} Treg, Foxp3^{egfp-}GATA3^{hDC2+} Th2 cell, ILC2 and common lymphoid progenitor (CLP) was compared. Shown is a Venn diagram of transcripts expressed in each cell population (> 10 RPKM).

I) ST2 expression was measured on lung ILC2, GATA3^{+} and GATA3^{-} Treg and Th2 cells in WT mice on day 5 after treatment with IL-33 (i.n., day 0 and 1).

J) The indicated populations from mouse lungs (see **Fig S1A**) were analysed on day 5 for the expression of ST2 and intracellular Ki67, after treatment with IL-33 (i.n., days 0 and 1). ST2^{+} and ST2^{-} fractions were analysed for percentage of dividing (Ki67^{+}) cells.

K) WT mouse lung ST2^{+} and ST2^{-} Treg cells were stained for intracellular Ki67 expression on day 5 after IL-33 treatment (i.n., day 0 and 1).

L-M) Splenic CD4^{+} T cells from (CD45.1) WT and (CD45.2) *Il1rl1*$^{-/-}$ mice were enriched, and injected in to *Rag2*$^{-/-}$ mice at a 50:50 ratio (i.v.). These recipients were treated with PBS or IL-33 (i.n., days 0 and 1), followed by quantification of Ki67^{+} CD45.1 and CD45.2 CD4^{+} Foxp3^{+} Treg cells in the lungs on day 5 (IL-33 treated shown, **L**). The ratio of Ki67^{+} lung Treg cells in *Il1rl1*$^{-/-}$ over WT was calculated to establish if one had a selective advantage to proliferate in either PBS or IL-33 treated animals **(M)**.

Bar graphs indicate mean (± S.E.M). (**A**, general representation of gating), (**B**, n=4,4 at each time point, mean ±S.E.M., 2 repeat experiments), (**C**, n=4,4,4 left to right, ANOVA, 2 repeat experiments), (**E**, n=4,4,4,4 left to right, 3 repeat experiments), (**F**, n=4,4,4,4 left to right, ANOVA, 3 repeat experiments), (**G**, general representation of gating), (**H**, n=2, single experiment), (**I-K**, n=4 in all, general representation of gating shown, 2 repeat experiments), (**L-M**, n=5,5 left to right, two-tailed Student's t-test, 2 repeat experiments), ** = p≤0.01, *** = p≤0.001, **** = p≤0.0001.

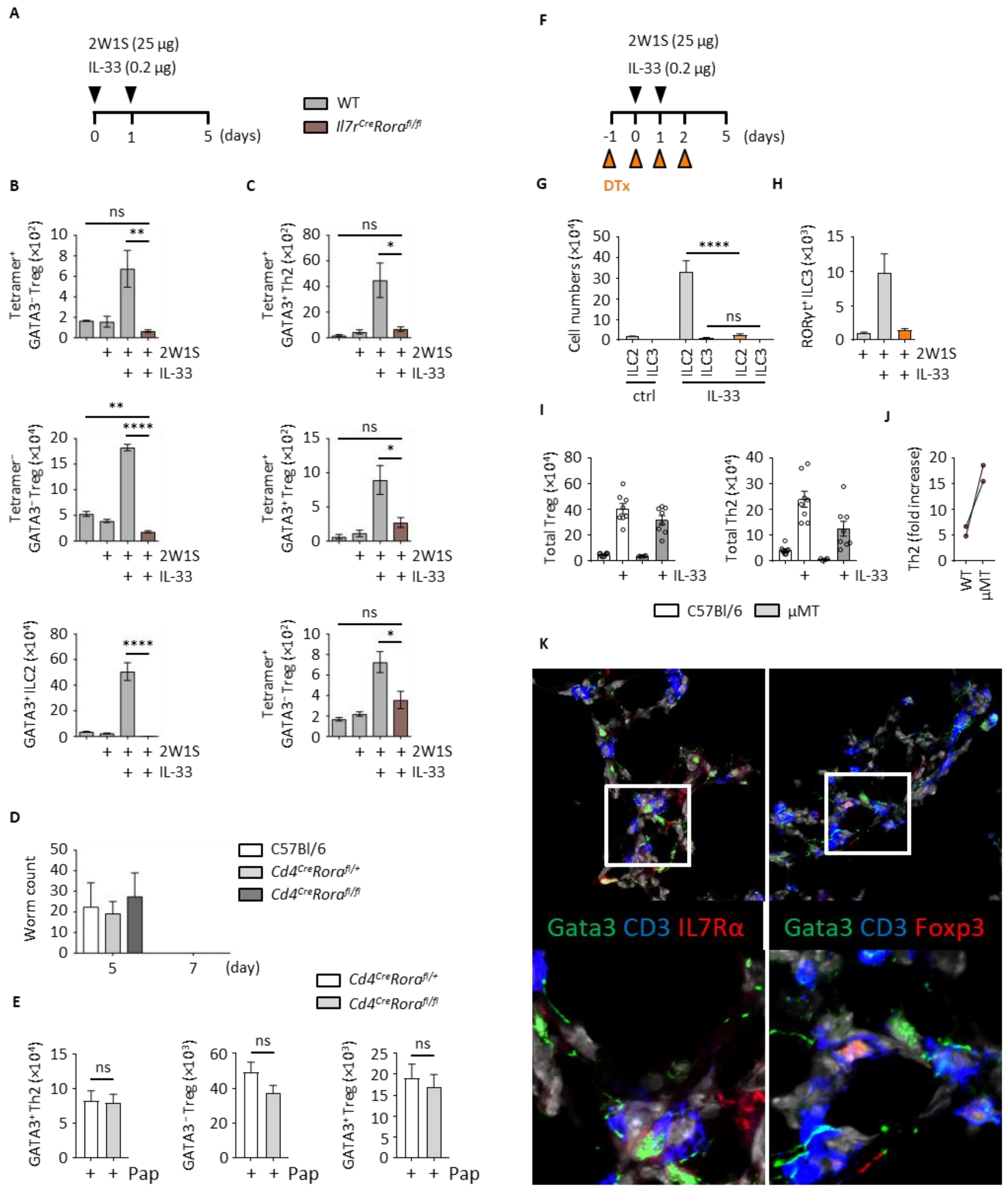

Figure S2 (Related to Figure 1)

A-C) WT and ILC2-deficient mice (*Il7r^Cre^Rora^fl/fl^*) were injected with 2W1S-peptide and IL-33 as indicated (i.n., day 0 and 1). Lung and mLN cells were collected on day 5, followed by analysis and quantification of 2W1S:Tetramer$^+$ and 2W1S:Tetramer$^-$ CD4$^+$ T cell populations and lung ILC2 (**B**) and mLN (**C**)

D) Mice of the indicated genotypes were infected with *Nippostrongylus brasiliensis* (500 L3 larvae, subcutaneously, day 0) followed by quantification of worms in the small intestines on day 5 and 7.

E) Mice of the indicated genotypes were injected with papain (Pap) (i.n., day 0, 1 and 22 or 23). Lung cells were collected on day 27 or 28, followed by analysis and quantification of Th2, GATA3^{+} Treg, and GATA3^{-} Treg cell populations.

F-H) ILC2-depleted (DTx) and non-depleted ICOS-T (PBS) mice (Oliphant et al., 2014) were injected with 2W1S-peptide and IL-33 as indicated (i.n., day 0 and 1). Lung and mLN cells were collected on day 5, followed by analysis and quantification of 2W1S:Tetramer^{+} and 2W1S:Tetramer^{-} CD4^{+} T cell populations and lung ILC2 and Live CD45^{+}B220^{-}CD3ε^{-}CD4/8^{-}NK1.1^{-}Lin^{-}CD127^{+}GATA3^{-}RORγt^{+} ILC3 (**G and H**).

I-J) Mice of the indicated genotypes were injected with IL-33 on days 0 and 1, followed by analysis on day 5 of total lung Treg and Th2 cell number (**I**), and the fold increase of Th2 cells in each genotype (**J**). Average fold increase is shown for two independent experiments.

K) Frozen sections from IL-33 treated (i.n., day 0 and 1) mouse lungs, collected on day 5, were stained for CD3, GATA3, and IL7Rα or Foxp3 as indicated. Bottom panel shows the gated region in the upper panel. Scale bar denotes 50 µm. Data are representative of at least 2 independent experiments.

Bar graphs indicate mean (± S.E.M). (**B-C**, n=4,4,4,4 left to right, ANOVA, 3 repeat experiments), (**D**, n=4,7,6,4,7,6 left to right, ANOVA, 2 similar repeat experiments), (**E**, n=10,10 left to right for all panels, two-tailed Student's t-test, 2 pooled experiments shown), (**G**, n=4,4,4 (for both ILC2 and ILC3) left to right, ANOVA, 3 repeat experiments), (**H**, n=4,4,4 left to right, 3 repeat experiments), (**I**, n=8,8,8 left to right, ANOVA, pooled data of 2 repeat experiments), (**J**, data points represent mean fold increase per genotype, 2 independent experiments), ns = not significant, * = p≤0.05, ** = p≤0.01, **** = p≤0.0001.

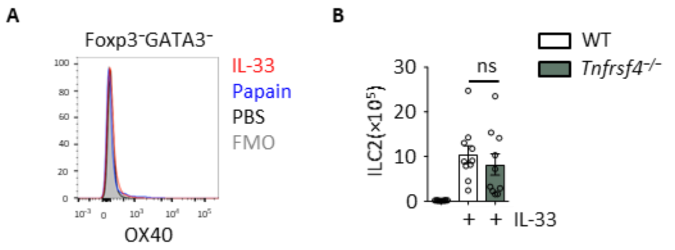

Figure S3 (Related to Figure 2)

A) OX40 expression was analysed on CD4^{+} Foxp3^{-}GATA3^{-} DN T cells from mice treated with PBS, papain (Pap) or IL-33. Fluorescence minus one (FMO) control was used to establish gating threshold.

B) Lung ILC2 cells were quantified on day 5 post IL-33 treatment (i.n.) in PBS and IL-33 treated WT and *Tnfrsf4*$^{-/-}$ mice.

Bar graphs indicate mean (± S.E.M). (**A**, general representation of gating), (**B**, n=10,10,10 left to right, ANOVA, 2 pooled repeat experiments), ns = not significant.

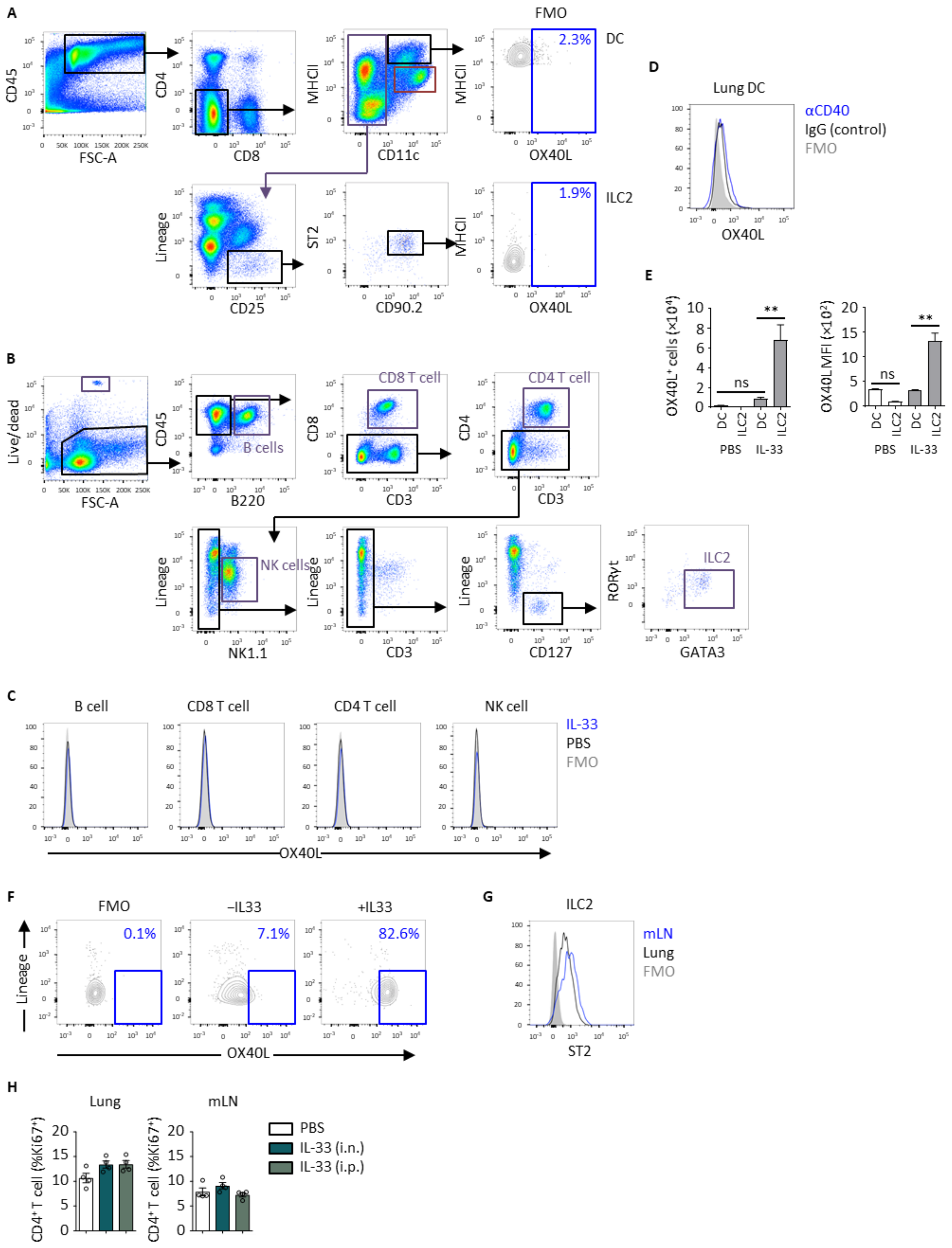

Figure S4 (Related to Figure 3)

A-B) OX40L expression on different immune cells was established by flow cytometry analysis. Lung cells from WT mice were gated for $CD45^+$ leukocytes and dead cells were excluded (not shown), followed by the indicated gating strategies to identify dendritic cells (DC), macrophages (MΦ), ILC2, ILC3, natural killer (NK) cells, B cells, CD8 and CD4 T cells. Fluorescence minus one (FMO) controls were used to set the gate for OX40L expression.
C) OX40L expression was measured on the indicated immune cells in WT mice on day 2 after stimulation with PBS or IL-33 (i.n., day 0 and 1).
D) Mice were administered anti-CD40 (αCD40) or IgG control mAb on days 0 and 1, followed by analysis of lung DCs for OX40L expression on day 2.
E) The absolute number of $OX40L^+$ ILC2 and DC in the lungs was calculated in PBS or IL-33 stimulated mice on day 3 (left panel). Mean fluorescence intensity (MFI) of OX40L staining was calculated for ILC2 and DC in the lungs of PBS or IL-33 stimulated mice on day 3 (right panel).
F) Human blood ILC2 were purified by FACS, followed by stimulation in culture with IL-2 and IL-7 (20 ng/ml) ± IL-33 (20 ng/ml) for 3 days. On day 3, cells were analysed for expression of OX40L on $CD45^+lineage^-$ ILC2.
G) ST2 expression was analysed on mLN and Lung ILC2 ($CD45^+B220^-CD4^-CD8^-CD3^-NK1.1^-Lineage^-CD25^+GATA3^+$) in mice treated as in **Fig 3E**. Fluorescence minus one (FMO) control was used to indicate background staining.
H) WT mice received IL-33 via intranasal or intraperitoneal route (day 0 and 1, PBS control), followed by analysis (day 2) of lung and mLN $\%Ki67^+$ $CD4^+$ T cells.

Bar graphs indicate mean (± S.E.M). (**A-C**, general representation of gating, 2-3 repeat experiments), (**D**, representative histogram, 2 repeat experiments), (**E**, n=3,3,3,3 left to right, ANOVA, 2 repeat experiments), (**F**, single individual sorted ILC2 exposed to different *in vitro* conditions shown, percent gated populations shown, 2 repeat experiments), (**G**, representative histogram, 3 repeat experiments), (**H,** n=4,4,4 left to right, ANOVA, 2 repeat experiments), ns = not significant, ** = p≤0.01.

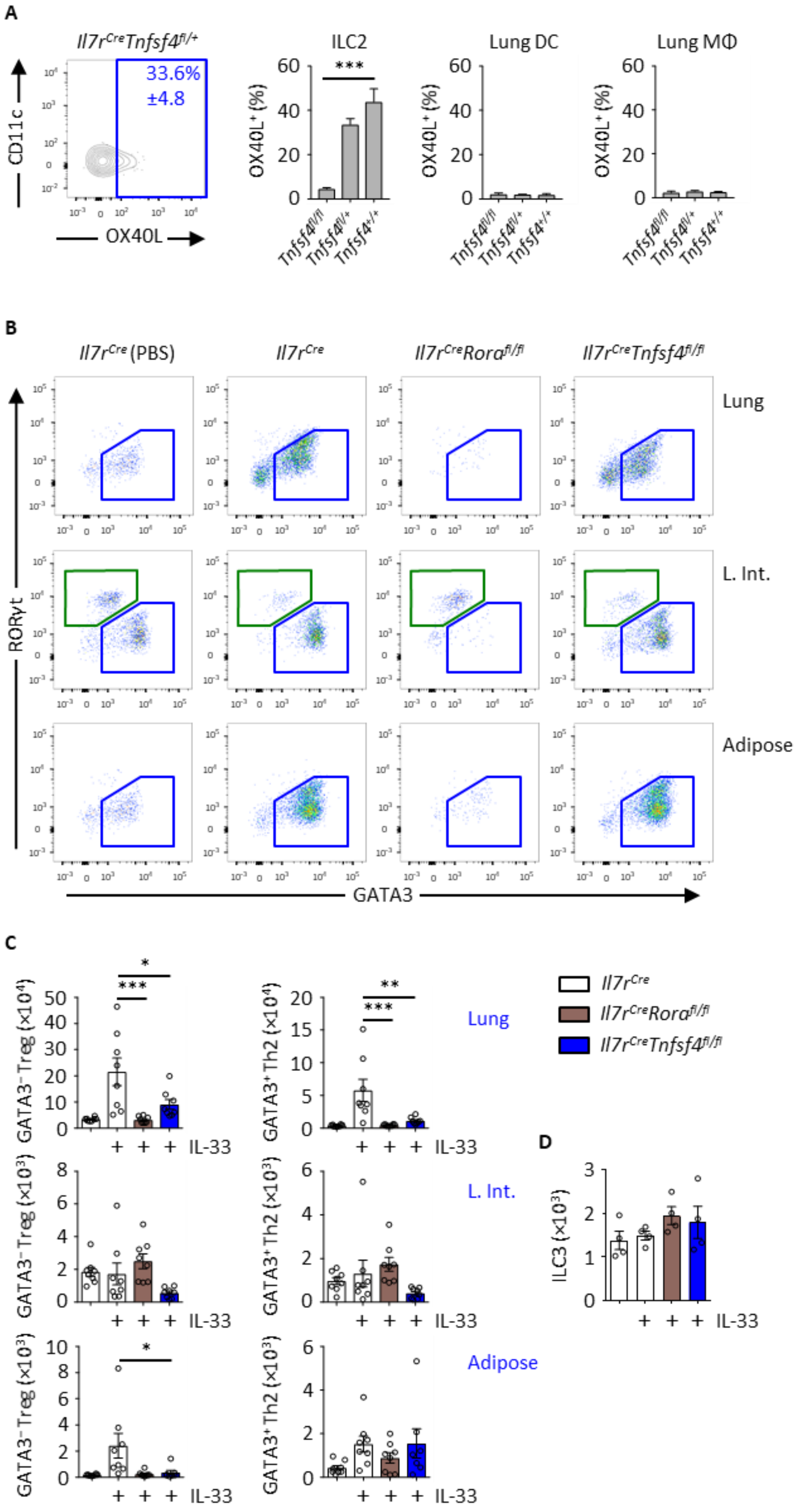

Figure S5 (Related to Figure 4)

A) OX40L expression was measured on lung ILC2, DC and MΦ from *Il7r*^{Cre}, *Il7r*^{Cre}*Rora*$^{fl/fl}$ and *Il7r*^{Cre}*Tnfsf4*$^{fl/fl}$ mice on day 2 after IL-33 administration (i.n., day 0 and 1). FMO control was used to establish gating threshold.

B) Mice of the indicate genotypes were analysed for ILC2 (blue) and ILC3 (green) in the indicated tissues (L. int., large intestine) on day 5 (as in **Fig S1A**) after treatment with PBS or

IL-33 (i.p., day 0 and 1). Total ILC2 were quantified, and are displayed on the right. Adipose cell numbers are normalised to cells/mg of tissue.

C-D) Mice of the indicate genotypes were analysed for GATA3⁻ Treg and Th2 cells in the indicated tissues on day 5 (as in **Fig S1A**) (**C**), and ILC3 (**D**) after treatment with PBS or IL-33 (i.p., day 0 and 1).

Bar graphs indicate mean (± S.E.M). (**A**, right panel shows percent gated ± S.D., n=4, left bar graphs n=4,3,4 left to right, 2 repeat experiments). (**B**, right panel shows general representation of gating, 2 repeat experiments). (**C**, n=8,8,8,8 left to right, ANOVA, 2 pooled repeat experiments, (n=8,8,8,7 for Adipose)), (**D**, n=4,4,4,4 left to right, 2 repeat experiments), * = p≤0.05, ** = p≤0.01, *** = p≤0.001.

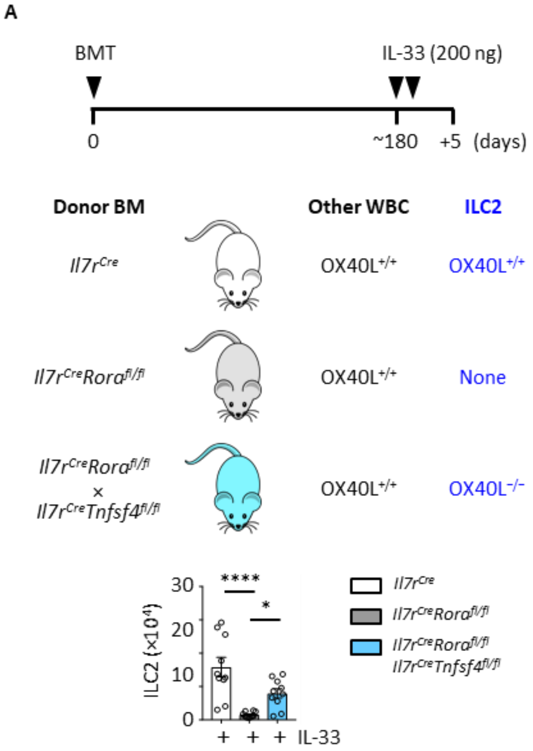

Figure S6 (Related to Figure 5)

A) Bone marrow and mixed-bone marrow (BM) chimeric animals were created with the indicated genotypes. Bone marrow transfer (BMT) was performed on day 0 after lethal irradiation (9.0 Gy). 6 to 7 months after bone marrow transfer, mice received IL-33 (i.n., days 0 and 1) followed by quantification of lung ILC2 cells on day 5.

Bar graphs indicate mean (± S.E.M). (**A**, n=10,10,10 left to right, ANOVA, 2 pooled repeat experiments), * = p≤0.05, **** = p≤0.0001.

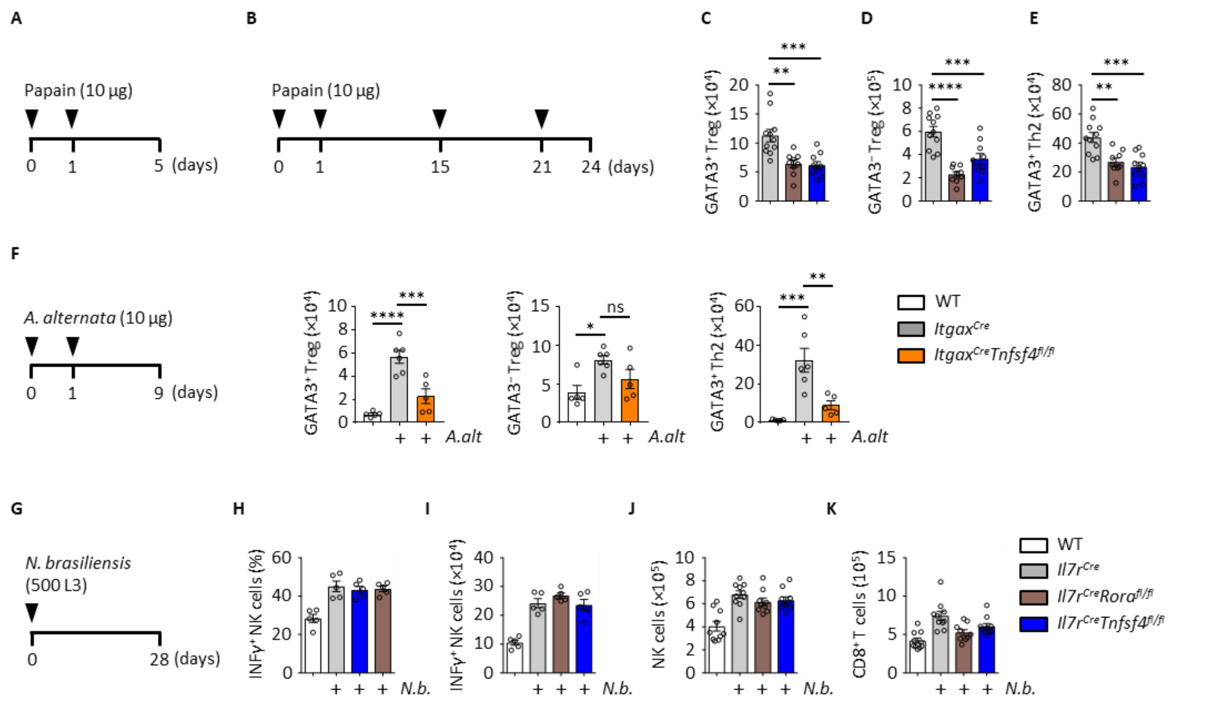

Figure S7 (Related to Figure 6 and 7)

A-B) Mice were treated with PBS or papain (Pap) as indicated, followed by analysis on day 5 or 24.

C-E) Mice of the specified genotypes were treated with papain (Pap) on days 0, 1, 14 and 21, followed by quantification on day 24 of lung Th2, GATA3$^+$ Treg, and GATA3$^-$ Treg cells.

F) Mice of the specific genotypes were injected with *A. alternata* (*A.alt*) on days 0 and 1 (i.n.), followed by analysis on day 9 of lung Th2, GATA3$^+$ Treg, and GATA3$^-$ Treg cells.

G) Mice of the specified genotypes were infected with *Nippostrongylus brasiliensis* (*N.b.*) on day 0, followed by analysis on day 28 of:

(H-I) Whole lung cell-suspensions were re-stimulated with PMA and ionomycin, followed by identification of IFNγ$^+$ NK cells by intracellular staining. Percent and total IFNγ$^+$ NK cells positive cells are shown.

J) Total lung NK cells numbers were quantified.

K) Total CD8$^+$ T cell numbers were quantified.

Bar graphs indicate mean (± S.E.M). (**C-E**, n=11,9,10 left to right, ANOVA, 3 repeat experiments (2 pooled experiments shown)), (**F**, n=5,5,5 left to right, 2 repeat experiments), (**H** and **I**, n=5,5,5,5 left to right, ANOVA, 2 repeat experiments), (**J** and **K**, n=10,10,10,10 left to right, ANOVA, 3 repeat experiments (2 pooled experiments shown)), ns = not significant, * = p≤0.05, ** = p≤0.01, *** = p≤0.001, **** = p≤0.0001.
